# Drug-induced death of leukaemic cells after G2/M arrest: higher order DNA fragmentation as an indicator of mechanism.

**DOI:** 10.1038/bjc.1998.7

**Published:** 1998

**Authors:** R. J. Sleiman, D. R. Catchpoole, B. W. Stewart

**Affiliations:** Children's Cancer Research Institute, University of New South Wales, Sydney Children's Hospital, Australia.

## Abstract

**Images:**


British Journal of Cancer (1998) 77(1), 40–50
© 1998 Cancer Research Campaign

### Drug-induced death of leukaemic cells after $G_2$/M arrest: higher order DNA fragmentation as an indicator of mechanism

**RJ Sleiman, DR Catchpoole* and BW Stewart**

Children's Cancer Research Institute, University of New South Wales, Sydney Children's Hospital, Sydney 2031, Australia

**Summary** Many reports have documented apoptotic death in different cell types within hours of exposure to cytotoxic drugs; lower drug concentrations may cause cell cycle arrest at $G_2$/M and subsequent death, which has been distinguished from 'classic' apoptosis. We have analysed etoposide-induced cell death in two lymphoblastoid T-cell lines, CCRF-CEM and MOLT-4, specifically in relation to DNA cleavage as indicated by pulse-field gel and conventional electrophoresis. High (5 μM) concentration etoposide causes 50-kb cleavage of DNA that occurs at the same time as apoptotic morphology and internucleosomal cleavage. At lower concentrations (0.5–0.05 μM), sequential change may be discerned with altered gene expression being similar to that at high dose, but preceding cell cycle arrest and 50-kb cleavage. These last changes, in turn, clearly precede internucleosomal fragmentation of DNA, vital dye staining and morphological evidence cell death. The pattern of higher order fragmentation constitutes a sensitive indicator of commitment to cell death in these cells. Morphological evidence of cell death is associated with internucleosomal fragmentation in one of the lines, but the pattern of 50-kb DNA cleavage provides the clearest evidence of commonality in death processes occurring at low and high drug concentration.

**Keywords:** apoptosis; 50-kb DNA breakage; etoposide; leukaemia; $G_2$/M arrest

The term 'apoptosis' is reasonably equated with physiological cell death during immunological selection and biological development (Wyllie, 1987; Allen et al, 1993). It is also recognized that this mode of cell death is induced by certain markedly non-physiological stimuli exemplified by radiation and certain drugs and specifically including most agents used in cancer chemotherapy (Hickman, 1992; Sachs and Lotem, 1993; da Silva et al, 1996). In the laboratory, induction of apoptosis by drugs has facilitated identification of processes associated with cell death, and of these internucleosomal fragmentation of DNA has been pre-eminent (Barry et al, 1990; Stewart, 1994). This effect is induced by a multiplicity of agents thereby providing evidence of a common pathway considered to be initiated by drug–target interaction and to culminate in cell death (Lennon et al, 1991; Marks and Fox, 1991). Accordingly, internucleosomal fragmentation of DNA, usually detected as DNA ladders after conventional agarose gel electrophoresis, has been perceived as the hallmark of apoptosis (Compton, 1992). However, the biological significance accorded to internucleosomal fragmentation of DNA is changing from the perception of an early indicator (Barry and Eastman, 1992; Smith et al, 1992), to a molecular change concurrent with morphological evidence of cell death (Lazebnik et al, 1995) and denoted by Vaux and Strasser (1996) as a 'post-mortem' event. Moreover, some cell populations do not exhibit ladders after drug treatment (Bertrand et al, 1991; Hotz et al, 1992; Falcieri et al, 1993). Other studies, however, indicate the absence of ladders when cell death is not immediate, but, in response to lesser concentrations, occurs 24 h or more after the addition of the drug (Ormerod et al, 1994; Bonelli et al, 1996).

Many, perhaps most, studies of cell death induced by anti-cancer drugs have involved cell lines exposed to a sufficiently high drug concentration that has an immediate effect, i.e. cause cell death within 8 h or less. Both apoptic morphology and internucleosomal fragmentation of DNA may be evident, the term 'classic apoptosis' being used. Although cytotoxic drugs commonly cause $G_2$/M cell cycle arrest (Rao and Rao, 1976; Chow and Ross, 1987), the same agents, when used at concentrations that cause cell death within hours, result in $G_1$ arrest (Chen et al, 1995; Dou et al, 1995; An and Dou, 1996). $G_1$ arrest is intimately associated with functional p53 (Lowe et al, 1993), and the role of this gene in mediating drug-induced apoptosis is clear and has been implicated in determining response to therapy (Lowe et al, 1994; Eliopoulos et al, 1995; Rusch et al, 1995; Marks et al, 1996). However, in many instances, drug concentations in patient serum are less than those used in the laboratory. Thus, in common with others, we studied death of lymphoblastoid cells within hours of exposure to 5 μM (or more) etoposide (Catchpoole and Stewart, 1993); clinical concentrations of this drug are typically 0.05 μM and rarely exceed 0.5 μM (Mross, 1996). Different pathways to cell death, dependent upon drug concentration, have been discerned (Del Bino et al, 1991; Lock et al, 1994; Tornaletti and Pfeifer, 1995; Jordon et al, 1996). In experimental cell populations exposed to drug concentrations which cause $G_2$/M arrest, the mode of cell death has been perceived to be distinct from classic apoptosis and, in some such cases, the term mitotic death has been proposed (Chang and Little, 1991; Demarcq et al, 1994; Ormerod et al, 1994; Dou et al, 1995; Bonelli et al, 1996). We have sought to investigate this mode of

*Received 10 February 1997*
*Revised 10 June 1997*
*Accepted 11 June 1997*

*Correspondence to:* BW Stewart

**Present address:* Institute of Molecular and Cell Biology, National University of Singapore, 10 Kent Ridge Crescent, Singapore 119260.

cell death using two lymphoblastoid cell lines CCRF-CEM (CEM) and MOLT-4 that have been exposed to etoposide concentrations between one and two orders of magnitude less than that which causes immediate cell death. A particular focus was the emergent hypothesis that, during apoptosis, internucleosomal cleavage may be accompanied by higher order fragmentation of DNA.

Detection of DNA fragments of 50 kb or more by pulse-field gel electrophoresis (PFGE) after exposure of cells to etoposide was initially interpreted with reference to the location of topoisomerase II (Filipski et al, 1990). Subsequently, the same effect was associated with apoptosis caused by a variety of stimuli, including agents not associated with direct damage to DNA (Brown et al, 1993; Oberhammer et al, 1993; Cohen et al, 1994; Desjardins and MacManus, 1995).

In the present study, we monitored genomic fragmentation by both conventional and PFGE and examined its relationship to changes in gene expression after treatment with etoposide at both high (5 μM) and low (0.5–0.05 μM) concentration. Many cell types undergo differentiation when exposed to cytotoxic drugs at low concentration, and the contribution of induced differentiation to induction of programmed cell death remains uncertain (Warrell, 1997). Specifically in relation to T cells, progressive differentiation is associated with increased susceptibility to apoptosis and correlated with gradual loss of bcl-2 and gain of Fas expression (Salmon et al, 1994). T-cell differentiation is marked by sharp changes in the expression of purine metabolic enzymes including purine nucleoside phosphorylase (PNP) (Ma et al, 1983) and such change is induced by phorbol esters (Martinez-Valdez and Cohen, 1988). Consistent with the intimate relationship between normal and malignant lymphoid cells (Campana and Janossy, 1988), altered expression of genes associated with purine metabolism is induced in CEM cells by phorbol 12-myristate 13-acetate (PMA) (Madrid-Marina et al, 1990). In preliminary experiments, we compared PMA with etoposide and established that drug-induced changes in PNP and terminal deoxynucleotide phosphorylase preceded and were more marked than altered adenosine deaminase expression. On this basis, we have contrasted the effect of etoposide with that of PMA in relation to PNP expression and other parameters including morphological change and genomic fragmentation.

After exposure to etoposide at high concentration, changes in 50-kb cleavage of DNA, gene expression and cell cycle progression were all evident within 6 h and not only occurred simultaneously but also coincided with morphological change and altered membrane permeability. Experiments with etoposide at low concentration revealed a more informative scenario. Sequential change may be discerned, providing a means for establishing relationships between classic apoptosis and cell death after $G_2$/M arrest. In this context, 50-kb fragmentation of DNA is established as a sensitive indicator of commitment to the cell death/apoptotic pathway and may be clearly shown to precede internucleosomal fragmentation, which occurs coincident with morphological evidence of cell death.

### MATERIALS AND METHODS

### Cell biology

MOLT-4 and CEM cell lines were maintained in RPMI-1640 medium supplemented with 2 mM L-glutamine and 10% fetal bovine serum (Biosciences Pty, Australia) at 37°C. Cells in logarithmic growth were treated with etoposide (Sigma Chemicals, St Louis, MO, USA) in dimethyl sulphoxide (DMSO, final concentration, 0.05%), the vehicle alone being added to control cultures. Cell numbers were determined by phase-contrast light microscopy using a haemocytometer. Procedures for treatment and harvesting have been described (Catchpoole and Stewart, 1993).

### Morphological analysis

Cytospin preparations were made with 100-μl ($4 \times 10^5$ cells) aliquots and stained using Wright's solution (Catchpoole and Stewart, 1993). Morphological assessment was made by light microscopy. Quantification of specific lesions was made by examination of at least 150 cells per field from at least three separate experiments and the result was expressed as the mean percentage of cells exhibiting the structure specified.

### Reverse transcriptase polymerase chain reaction (RT-PCR)

Total cytoplasmic RNA was isolated (Chomczynski and Sacchi, 1987) and reverse transcribed using Maloney murine leukaemia virus DNA polymerase and random hexanucleotide primers (Noonan et al, 1990). Gene-specific primers for PNP (sense: 5′-ATGCAGCAGGAGGGCT-GAAC-3′; antisense 5′-AGGCATCAGACATGGCAGGG-3′; product length, 153 nucleotides) and glyceraldehyde-3-phosphate dehydrogenase (GAP) (sense: 5′-TGGGGAAGGTGAAGGTCGGA-3′; antisense 5′-TGGTGCAGGAGGCATTGCTG-3′; product length, 110 nucleotides) were used to co-amplify target mRNA sequences in a competitive PCR reaction essentially as described by Bordow et al (1994), including primer design to negate possible DNA contamination of the original isolate and the establishment of a cycle number corresponding to linear incorporation. A cDNA amount equivalent to 40 ng RNA was subjected to PCR for 35 cycles in a final volume of 25 μl using 1 unit of Ampli*Taq* Polymerase. The optimized reaction conditions involved an initial denaturation at 94°C: each cycle consisted of 45 s at 94°C, 45 s at 55°C and 90 s at 72°C. After PCR, aliquots (10 μl) were subjected to 12% polyacrylamide gel electrophoresis before ethidium bromide staining and photography. To quantitate product formation after competitive PCR, photographic negatives were densitometrically scanned (Hoeffer GSV300 Australian Chromatography, Sydney, Australia) and a ratio between target and control PCR products was determined for each sample.

### Flow cytometry

Cell cycle distribution was determined by flow cytometry after propidium iodide staining (Deitch et al, 1982), as described previously (Catchpoole and Stewart, 1993). Using red propidium–DNA fluorescence, 10 000 events were acquired with a FACScan cytometer and analysed using LYSIS II and Cellfit software (Becton Dickinson, San Jose, CA, USA).

### Agarose gel electrophoresis

Internucleosomal DNA breakage was detected using conventional agarose gel electrophoresis after isolation of DNA as follows. Cells ($2 \times 10^6$) were washed in phosphate-buffered saline (PBS) by centrifugation at 10 000 *g* at 4°C for 5 min. Cell pellets were lysed in 200 μl of DNAzol (Molecular Research Centre, Cincinnati, OH, USA) by gently pipetting the suspension. RNAase A was added

© Cancer Research Campaign 1998

**Figure 1** The relationship between etoposide concentration and growth of CEM and MOLT-4 cells. At time zero, etoposide at final concentrations of 0.05 μM (upright triangle), 0.5 μM (inverted triangle), 5 μM (circle) was added to the medium of CEM (closed symbols) or MOLT-4 cells (open symbols) and cell numbers for up to 48 h in the presence of the drug were contrasted with those in cultures receiving the vehicle alone (squares). At the highest etoposide concentration used, the numbers could not be determined beyond 24 h because of cellular debris and cytolysis

(30 μl of a 10 mg ml$^{-1}$ stock) and the suspension incubated for 30 min at 37°C and further centrifuged for 10 min at 10 000 ***g*** at 4°C. After centrifugation, the viscous supernatant was transferred to a fresh tube. DNA was precipitated by addition of 100 μl cold 100% ethanol and pelleted by centrifugation for 30 min at 10 000 ***g*** at 4°C. The resulting DNA precipitate was washed twice with 800 μl of 95% ethanol and allowed to air dry for 30 min at room temperature before resuspending in 80 μl of 8 mM sodium hydroxide buffered with 2 μl 1 M Hepes (*N*-2-hydroxyethylpiperazine-*N*′-2-ethanesulfonic acid; final pH 7.2) and subjected to electrophoresis in 1.8% agarose (Catchpoole and Stewart, 1993).

### PFGE

Cells (5 × 10$^{6}$) were resuspended in 60 μl of cell suspension buffer (10 mM Tris, pH 7.2, 20 mM sodium chloride, 50 mM EDTA) and allowed to equilibrate at 50°C. The suspension was gently mixed with 37 μl of pre-warmed (50°C) '2% Clean Cut Agarose' (Bio-Rad, Hercules, CA) and the mixture immediately transfered to plug moulds (using sterile transfer pipettes) and incubated at 4°C for 20 min to expedite agarose solidification. Agarose plugs were then subjected to proteinase K digestion by the addition of 500 μl of 100 mM EDTA, pH 8.0, 0.2% sodium deoxycholate, 1% sodium lauryl sarcosine solution containing 40 μg ml$^{-1}$ proteinase K and incubated at 50°C overnight. Plugs were washed for 1 h (× 4) in wash buffer (20 mM Tris, pH 8.0, 50 mM EDTA) at room temperature with gentle agitation and stored in 0.1 × wash buffer at 4°C before electrophoresis. Samples, including a DNA molecular weight standard (48.5 kb lambda ladder, Bio-Rad), were electrophoresed through 1% pulsed field gel grade agarose in a horizontal electrophoresis system (model CHEF-DR-III; Bio-Rad Laboratories). The run parameters included a ramped switch time of 50–90 s over 22 h at 6 V cm$^{-1}$ across an included angle of 120° in a 0.5 × TBE (89 mM Tris-HCl, 89 mM sodium borate, 2 mM EDTA pH 8.3) buffer system. The buffer was allowed to recirculate continuously and the temperature maintained at 14°C using a cooling module. After staining with ethidium bromide (0.5 μg ml$^{-1}$) for 30 min, the gels were photographed under UV irradiation.

### RESULTS

We have previously examined the response of CEM and MOLT-4 cells to etoposide in terms of internucleosomal fragmentation of DNA, cell cycle distribution and morphological change (Catchpoole and Stewart, 1993; 1995). A feature of these data was the difference between the two cell populations. In studies using 5–100 μM etoposide, CEM cells exhibited 'classic' apoptosis, whereas internucleosomal fragmentation and apoptotic bodies were not evident in the MOLT-4 population. Moreover, although the drug was equally cytotoxic in both lines, loss of membrane integrity appeared to have different kinetics. Despite these differences, there was a uniformity in response of both the CEM and the MOLT-4 populations: exposure to etoposide caused immediate apoptotic cell death as determined by morphological criteria. The response was independent of drug concentration: if this were reduced from 100 μM to 5 μM, apoptosis was still evident within 6 h. The present data indicate that this is not the case if the etoposide concentration is reduced by one further order of magnitude. Whereas exposure to 0.5 μM etoposide causes cell death in both CEM and MOLT-4 cells, this lesser concentration is associated with an altered response such that cell death is preceded by changes not evident using the high concentration range previously studied.

### Growth patterns

As determined by MTT cytotoxicity assay (72-h exposure), CEM and MOLT-4 cells are equally sensitive to etoposide, the $ID_{50}$ being approximately 0.05 μM (Catchpoole and Stewart, 1993). The determination of cell number up to 48 h after addition of the drug provided further evidence that the two lines were equally sensitive. In relation to an effect on cell number, no adverse effect was apparent in either population after the addition of 0.05 μM etoposide. Stasis in cell number over the incubation period was achieved using 0.5 μM etoposide (Figure 1) and a sharply decreased number of cells was evident after the addition of 5 μM etoposide.

### Morphology

Light microscopic examination was made of cells exposed to either etoposide or PMA. In the case of the phorbol ester, no treatment-related changes were apparent in CEM (Figure 2A and E) or MOLT-4 cells (data not shown). Morphological evidence of cell death is obvious 6–12 h after exposure of CEM cells to 5 μM etoposide (Figure 2F). In contrast, light microscopic examination of cells exposed to 0.5 μM or 0.05 μM etoposide for up to 24 h revealed minimal evidence of injury and cell death. There was progressive accumulation of darkly staining nuclei indicative of dividing cells after the addition of etoposide. In addition, the drug-treated cells could be distinguished from appropriate controls because cytoplasmic vacuoles were more common in cells exposed to etoposide (Figure 2A–C and G). When incubation was continued from 24 to 48 h, a marked change was immediately obvious. Cell death and associate debris were prominent to the point of obscuring any unaffected cells. Lesions specifically associated with apoptosis were evident: condensed chromatin, apoptotic bodies and secondary necrosis (Figure 2D and H). Multipolar mitotic figures

© Cancer Research Campaign 1998

**Figure 2** The appearance of CEM cells after exposure to etoposide and PMA. Wright-stained cytospin slide preparations were prepared after exposure to 0.5 μM etoposide for 12 (B), 24 (C) and 48 h (D), 5 μM etoposide for 12 h (F) and to 0.05 μM etoposide for 24 (G) and 48 h (H). Etoposide-induced morphological change may be discerned by comparison with vehicle control cells (0.05% DMSO for 24 h; A) and cells exposed to 10 nM PMA for 12 h (E). Morphological features evident include mitosis (M), cytoplasmic vacuoles (CyV) as well as change specifically indicative of cell death, including cytoplasmic protrusions (CyPr), apoptotic bodies (ApB), condensed chromatin (Ccr), primary necrosis (1°N) and secondary necrosis (2°N), these terms identifying lysis of whole cells and apoptotic bodies respectively

© Cancer Research Campaign 1998 

**Table 1** Quantification of morphological change induced by 0.5 μM etoposide

| Treatment time (h) | Cytoplasmic vacuoles | Cytoplasmic protrusins | Marginated condensed chromatin | Secondary necrosis |
|---|---|---|---|---|
| CEM | | | | |
| Vehicle control | 4.0 ± 0.4[a] | 1.6 ± 0.6 | 0.9 ± 0.2 | 4.2 ± 1.5 |
| 3 | 8.4 ± 1.9 | 3.3 ± 1.0 | 1.6 ± 0.6 | 2.4 ± 0.4 |
| 6 | 8.8 ± 0.96 | 5.1 ± 0.6 | 3.1 ± 0.9 | 3.8 ± 1.8 |
| 12 | 7.5 ± 0.6 | 6.0 ± 0.8 | 4.2 ± 1.5 | 2.4 ± 0.6 |
| 24 | 21.1 ± 6.1 | 5.3 ± 1.0 | 4.7 ± 2.1 | 5.1 ± 1.8 |
| 48 | 81.3 ± 3.1 | > 90[b] | > 90 | 55.3 ± 2.8 |
| MOLT-4 | | | | |
| 24 | 16 ± 3.1 | 6.0 ± 1.0 | 1.1 ± 0.5 | 2.5 ± 0.9 |
| 3 | 5.8 ± 1.7 | 3.8 ± 0.9 | 3.1 ± 1.5 | 2.9 ± 1.6 |
| 6 | 6.4 ± 0.6 | 3.1 ± 1.2 | 3.3 ± 1.4 | 1.3 ± 1.0 |
| 12 | 7.3 ± 1.4 | 5.1 ± 1.6 | 6.9 ±1.6 | 4.9 ± 1.2 |
| 24 | 12.4 ± 1 | 6.7 ± 1.4 | 12.7 ± 2.7 | 3.8 ± 0.60 |
| 48 | > 90 | > 90 | > 90 | 18.7 ± 2.5 |

[a]The percentage of cells exhibiting each lesion was quantified as described in Materials and methods. [b]This result indicates that the result could not be precisely quantified because the majority of cells exhibited the lesion and cell debris precluded quantification.

were not seen. Cell debris limited detailed analysis and there was little distinction between preparations exposed to 0.5 and 0.05 μM etoposide. Essentially the same results were observed in similar preparations of MOLT-4 cells (data not shown).

Quantification of cells exhibiting various morphological lesions characteristic of apoptosis established that exposure of either CEM or MOLT-4 cells to 0.5 μM etoposide for up to 24 h rarely caused cell death. After such treatment, the number of cells containing vacuoles was increased, but no increase in the number of non-viable cells was evident in MOLT-4 or CEM preparations exposed to 0.5 μM etoposide for 24 h (Table 1). By comparison, the radical change in morphology consequent upon exposure of these cells to 0.5 μM etoposide for 48 h effectively precluded summation of discrete lesions: cell debris and secondary necrosis obscured many of the remaining cells, limiting quantitation to estimates only (Table 1).

### Gene expression

Increased expression of PNP was evident 6 h after exposure of CEM or MOLT-4 cells to 10 nM PMA. By the same criterion, i.e. quantification relative to expression of GAP using RT-PCR (Figure 3), a marked increase (greater than three-fold in terms of the arbitary units used) in the expression of PNP was recorded in CEM cells exposed to 0.5 μM etoposide and this increase was sustained till at least 12 h after the addition of the drug. Similar results were recorded using MOLT-4 cells (data not shown). When the concentration of etoposide was reduced to 0.05 μM, a similar induction of PNP occurred. Moreover, such altered PNP expression could not be distinguished from that occurring in cells exposed to 5 μM etoposide (Figure 3).

### Cell cycle

In contrast to the pattern of similarly increased expression of the marker gene PNP over the dose range 0.05–5 μM etoposide, a sharp demarcation was apparent when flow cytometry was used to assess cell cycle distribution. Earlier studies had involved 5 μM etoposide, and indicated that 3 or 6 h after the addition of the drug to the medium there was a slight increase in the proportion of $G_1$ cells. Even when such cultures were analysed after 24 h, cell cycle distribution within the small fraction of surviving cells exhibited no increase (relative to vehicle-treated controls) in the proportion of cells at $G_2$/M. In contrast, etoposide in the concentration range 0.5 μM–0.05 μM resulted in cell cycle arrest such that by 24 h the proportion of cells at $G_2$/M was three- to fivefold greater than in the untreated population (Figure 4). No change in cell cycle distribution of either CEM or MOLT-4 cells was evident after the addition of 0.005 μM etoposide.

### Genomic fragmentation

PFGE was used to examine higher order fragmentation of DNA from CEM and MOLT-4 cells exposed to PMA and etoposide. In addition to causing differentiation (Madrid-Marina et al, 1990), the exposure of leukaemic cells to PMA has been reported to cause internucleosomal fragmentation of DNA (Ohta et al, 1995). In our studies, no higher order fragmentation of DNA was evident by PFGE using DNA isolated from CEM or MOLT-4 cells exposed 10 nM PMA or after exposure to DMSO at vehicle–control concentration. However, such fragmentation was detected in DNA isolated from either cell line after the addition of etoposide to the media. Specifically, in preparations from MOLT-4 cells, broad bands corresponding to approximately 50–150-kb were observed 6 h after the addition of 5 or 50 μM etoposide although, as previously described, internucleosomal fragmentation of DNA was not detected under the same conditions.

Over the broad etoposide concentration range studied (50–0.05 μM), PFGE data involved bands of various intensity, but corresponding to a limited size range. Most bands were 50 kb, but variation to 150 kb occurred. This was common to both lines. No consistent pattern for this slight variation in size range was apparent: no progression from 150 kb to 50 kb with increasing treatment time was recorded, and no progression over the 50–150-kb range within increasing or decreasing concentration of etoposide could be discerned. Moreover, repeat experiments indicated that identical

 © Cancer Research Campaign 1998

**Figure 3** RNA isolated from control and drug-treated CEM cell preparations was reverse transcribed and subject to competive RT-PCR analysis as previously described (Bordow et al, 1994). (A) A representative RT-PCR gel showing changes in PNP expression (upper band) after 0.5 μM etoposide treatment in CEM after 3, 6, 12, 24 and 48 h exposure (lanes 4 to 8 respectively) compared with vehicle control (lane 1). Preparations corresponding to phorbol ester (10 nM PMA)-treated samples isolated after 6 and 12 h are in lanes 2 and 3 repectively. After densitometric analysis of the PCR products, the relative intensity shown is the ratio between the target gene PNP and the housekeeping gene GAP (lower gel band). (B) Progressive change in expression of PNP after treatment of CEM cells with 0.05 μM (■), 0.5 μM (◆) and 5 μM (●) etoposide. Each point is the mean relative expression based upon at least two experiments. The inset shows relative changes in PNP expression after 10 nM PMA treatment

**Figure 4** The proportion of cells at $G_2$/M after 24 h continuous exposure to etoposide. After drug treatment, CEM (■) and MOLT-4 cells (▨), cell cycle distribution was determined by flow cytometry and the proportion of $G_2$/M expressed as a percentage. Typical flow cytometric profiles, specifically involving 6 h exposure to 5 μM, 17 μM and 100 μM etoposide have been published (Catchpoole and Stewart, 1993). Results shown are the means of at least two experiments, except for 0.005 μM etoposide which records a single study treatment conditions involved fluctuation within the size range indicated. Such variation is often (Cohen et al, 1994; Zhivotovsky et al, 1994; Weis et al, 1995) but not invariably (Brown et al, 1993; Oberhammer et al, 1993; Beere et al, 1995) seen in similar PFGE analyses.

When the various etoposide concentration/exposure time combinations were assessed, the results from CEM and MOLT-4 cells were similar (Figure 5). After exposure to etoposide at high concentration (5 or 50 μM), 50–150-kb fragmentation of DNA was evident. Such breakage was detected 6 h after the addition of the drug and a similar result was recorded up to 24 h after the addition of 5 μM etoposide. It was not possible to make PFGE analysis at such times after 50 μM etoposide because complete cellular destruction precluded recovery of DNA. Such 50-kb breakage of DNA was not evident in preparations isolated 3 or 6 h after the addition of 0.5 or 0.05 μM etoposide. It was, however, consistently detected in DNA isolated 12 h, and most markedly observed in DNA isolated 24 h after the addition of the drug at these concentrations (Figure 5). These findings were equally clear in both MOLT4 and CEM cells.

Data from multiple PFGE studies are summarized in Table 2. Among other considerations, data from both cell lines indicate that results using a single exposure time would be inadequate to assess higher-order fragmentation of DNA caused by etoposide, the 6 h

**Figure 5** PFGE analysis of DNA from cells exposed to 0.05–50 μM etoposide. DNA from MOLT-4 (A) and CEM (B) cells was subject to PFGE and ethidium bromide fluorescence is presented as a photographic negative. For A, DNA was isolated from untreated cells (lane 1), cells exposed to etoposide for 3 h at concentrations of 0.05 μM, 0.5 μM and 5 μM (lanes 2–4), for 6 h at 0.05 μM, 0.5 μM and 5 μM (lanes 5–7), for 12 h at 0.05 μM and 0.5 μM (lanes 8 and 9). DNA was also isolated from cells exposed to 50 μM etoposide for 3 h and 6 h (lanes 11, 12). Preparations from CEM cells (B) correspond to untreated (lane 1), DMSO vehicle control for 24 h (lane 2), 10 nM PMA for 6 and 12 h (lanes 3, 4), 0.05 μM etoposide for 6, 12 and 24 h (lanes 5–7), 0.5 μM etoposide for 12, 24 and 48 h (lanes 8–10) and 5 μM etoposide for 6 h (lane 11). Markers were run in lanes indicated (M), with the 50-kb marker band indicated on the left of each panel

**Table 2** Etoposide-induced 50 kb and internucleosomal cleavage in CEM and MOLT-4 cells

| | Etoposide concentration (μM) | | | |
|---|---|---|---|---|
| **Exposure time** | **0.05** | **0.5** | **5** | **50** |
| CEM | | | | |
| 3 | –[a] | – | ± | ±(L) |
| 6 | ± | ± | +(L)[b] | ++(L) |
| 12 | + | + | ++(L) | ~~DNA~~[c] |
| 24 | ++ | ++ | ++(L) | ~~DNA~~ |
| 48 | – | ++(L) | ~~DNA~~ | ~~DNA~~ |
| MOLT-4 | | | | |
| 3 | – | – | – | + |
| 6 | – | – | + | ++ |
| 12 | + | + | ++ | ~~DNA~~ |
| 24 | + | ± | ~~DNA~~ | ~~DNA~~ |
| 48 | Not done | ++ | ~~DNA~~ | ~~DNA~~ |

[a]Summary results of multiple PFGE of DNA from cells exposed to etoposide are summarized using ++ to indicate the most intense band for the drug concentration used, + for consistent bands of lesser intensity, ± for results in which 50-kb bands were observed in some but not all analyses of DNA for the exposure time/etoposide concentration indicated and – to indicate non-detection of 50-kb breakage. Each result is indicative of at least two, and usually three or more analyses involving separate experiments. [b]DNA isolated from the same cells was subjected to conventional agarose gel electrophoresis and (L) indicates those protocols causing ladders; for all other samples, internucleosomal fragmentation was not detected. ~~DNA~~[c] indicates non-availability of DNA consequent upon complete cellular destruction after prolonged exposure to the drug at high concentration.

**Figure 6** Analysis of etoposide-induced internucleosomal DNA fragmentation by 1.8% agarose gel electrophoresis. DNA was isolated from CEM cells after 24 h exposure to 0.05% DMSO (lane 1), 12 and 24 h exposure to 0.2 μM etoposide (lanes 2 and 3); 12, 24 and 48 h exposure to 0.5 μM etoposide (lanes 4–6) and after exposure to 5 μM etoposide for 12 and 24 h (lanes 7 and 8). Internucleosomal fragmentation formation is evident in lanes 6–8. The corresponding analyses using DNA isolated from MOLT-4 cells revealed no evidence of internucleosomal fragmentation time point, for example, being appropriate to high concentrations but failing to detect positive results from lower drug concentrations.

Whereas PFGE analysis of DNA from CEM and MOLT-4 cells yielded similar results, the two lines differ most markedly in

**Figure 7** Cell death, as indicated by trypan blue staining, of CEM cells after continuous exposure up to and including 48 h to 0.005–50 μM etoposide. Results are expressed as the means of at least two studies. Similar results were obtained using MOLT-4 cells relation to internucleosomal fragmentation. We have described the absence of DNA ladders after etoposide treatment of MOLT-4 cells (Catchpoole and Stewart, 1993). Under the same conditions (i.e. high etoposide concentration), the occurrence of internucleosomal fragmentation in DNA from CEM cells is coincident with 50 kb breakage, i.e. it is evident 3–6 h after treatment. In contrast, DNA ladders were not detected 3, 6, 12 or 24 h after treatment with 0.5 μM etoposide, but were evident in DNA isolated 48 h after such treatment (Figure 6). Ladders were not detected in any DNA preparations from cells exposed to 0.05 μM etoposide. To facilitate direct comparison with PFGE data, these findings have been included in the section of Table 2 corresponding to CEM cells.

### Trypan blue

Delayed loss of membrane integrity is a recognized characteristic of apoptosis. In common with many of the other biological parameters studied, staining of CEM or MOLT-4 cells exposed to etoposide over a wide concentration range indicated two response patterns. At high etoposide concentrations (50 or 5 μM), approximately one-quarter of CEM cells were stained after 6 h, with the entire treated population showing uptake by 24 h. Staining of MOLT-4 cells after the same treatment was similar (i.e. immediately and progressively increasing), with all cells permeable to the dye by 48 h. A different response pattern was observed at lower drug concentrations. When etoposide concentration was decreased from 5 μM to 0.5 μM, a notable increase in trypan blue uptake was not evident before the 48-h time point, when 70% of cells were stained positive (Figure 7). A similar pattern was evident using 0.05 μM etoposide, although in this case only one-third of cells were killed by 48 h. Exposure of cells to 0.005 μM etoposide had no significant effect on viability by this criterion. Trypan blue staining of MOLT-4 cells exposed to 0.5 μM or lower concentration of etoposide yielded a result (data not shown) virtually identical to that recorded for CEM cells. In particular, positive staining of MOLT-4 cells exposed to 0.5 μM etoposide was only observed in the majority of the population after 48 h exposure, and not at earlier times.

© Cancer Research Campaign 1998

### DISCUSSION

Despite the use of DNA ladders as a 'hallmark' of apoptosis, this lesion is not observed during apoptosis induced by a variety of stimuli (Barres et al, 1992; Oberhammer et al, 1922), and specifically including cytotoxic drugs in some situations (Bertrand et al, 1991; Hotz et al, 1992; Catchpoole and Stewart, 1993; Falcieri et al, 1993). Although it is generally accepted that cytotoxic drugs elicit apoptosis via a 'final common pathway', there have been few molecular criteria apart from DNA ladders to characterize this process (Lennon et al, 1991; Marks and Fox, 1991). Relatively recently, however, higher-order fragmentation of DNA as evidenced by PFGE has been widely reported in cells during classic apoptosis (Bortner et al, 1995). Moreover, Hickman and colleagues (Beere et al, 1995) were able to distinguish between DNA breakage attributable to stabilization of topoisomerase II cleavable complex and higher-order fragmentation of DNA not associated with this mechanism. Not so readily summarized are findings on the relationship of higher-order breakage to internucleosomal fragmentation and to morphological change indicative of apoptosis. Our data, which contribute insight in relation to both these issues, were generated in the course of investigating whether a mode of cell death distinct from apoptosis could be characterized in lymphoblastoid cells exposed to etoposide at low concentration. The study was prompted by the observation that apoptotic morphology and internucleosomal fragmentation of DNA were not evident 6 or 24 h after incubation of CEM cells with 0.5 μM etoposide (Stewart et al, 1995), as distinct from positive findings using 5 μM, 17 μM or higher concentration etoposide (Catchpoole and Stewart, 1993). The findings appeared to support the conclusion reached by Ormerod et al (1994): namely that cisplatin-induced cell death of L1210 cells after $G_2$/M arrest should not be classified as apoptosis.

Whereas treatment of CEM and MOLT-4 with 5 μM etoposide caused cell death within hours (Catchpoole and Stewart, 1993) (see also Figure 2F) and a commensurate decrease in cell number, 0.5 μM etoposide caused a cytostatic effect (Figure 1). A growth response, involving murine fibroblast monolayers exposed to 0.1–10.0 μM etoposide, almost identical to that shown in Figure 1 was recently described by Bonelli et al (1996), although their study involved cell death at the two higher concentrations (i.e. 1.0 and 10 μM). In our study, histological evaluation indicated that despite the absence of any net decrease in total cell number (Figure 1), cell death occurred 48 h after exposure to 0.5 μM (or more) etoposide (Figure 2), the consistent cell number being attributable to some proliferative activity. Although a further decrease in drug concentration to 0.05 μM resulted in no effect on cell number by comparison with vehicle-treated controls, drug-induced cell death was again evident after light microscope examination (Figure 2).

Morphological evidence of cell death 48 h after exposure to 0.05 μM etoposide (Figure 2) indicates the limitation of cell count (Figure 1) as an immediate indicator of severe injury at low drug concentrations. Cell count does not differentiate between viable and non-viable (i.e. trypan blue positive) cells, the latter making up an increasing portion of the treated population (Figure 7). The data suggest that 48 h after the addition of 0.05 μM etoposide, dead and dying cells are still intact during phase contrast microscopy (for cell count), but rupture during cytospin preparation. Such fragmentation might give the appearance of necrosis despite the cell being commited to an apoptotic pathway.

The complex relationship between cellular proliferation, differentiation and apoptosis is indicated among other things by the differentiating effect of anti-cancer drugs at non-cytotoxic concentration (Constantinou et al, 1992; Schwartz et al, 1992; Li et al, 1995; Grant et al, 1996). Altered expression of genes associated with lymphocyte differentiation (Ma et al, 1983; Madrid-Marina et al, 1990) may have indicated a differential response in which lower etoposide doses were more closely associated with differentiation. However, the data (Figure 3) indicate that an immediately lethal concentration of etoposide (5 μM) caused an increase in PNP expression similar to that which occurred using concentrations 100-fold less. This response was similar to, if not more marked than that induced by PMA. In relation to etoposide concentration, similar induction of PNP precludes using this effect to identify different response pathways. Moreover, induction of PNP occurred within a similar time frame and there was no delay associated with lower concentrations of etoposide. Thus, a conspicuous feature of altered PNP expression is its immediacy. Of the biological parameters analysed (cell cycle progression, genomic fragmentation, dye uptake, etc.), monitoring gene expression revealed the earliest response to drug treatment.

After studying death of murine leukaemic cells induced by cisplatin, Eastman and colleagues (Sorenson et al, 1990) described two pathways: one involving a rapid, direct nuclease activation induced by very high drug concentrations and the other necessitating $G_2$/M arrest before cell death. This relationship appears to be common. Etoposide and similar agents have been variously reported to cause either $G_1$ (Chen et al, 1995; Dou et al, 1995; An and Dou, 1996) or $G_2$/M arrest (Rao and Rao, 1976; Chow and Ross, 1987), the difference being attributable, in the first instance, to drug concentration (Del Bino et al, 1991; Touneki et al, 1993). After the addition of 0.5 μM etoposide to CEM or MOLT-4 cells, the proportion of cells at $G_2$/M increased progressively in determinations made between 6 h and 24 h later, when a maximal value was recorded (data not shown). This response pattern involved a specific etoposide concentration range (Figure 4). However, although $G_2$/M arrest may precede drug-induced cell death in a variety of circumstances, the relationship between these effects varies. Thus, Schimke et al (1995), studying cell death after drug-induced $G_2$/M arrest in HeLa cells, describe aberrant mutipolar mitoses. However, we observed no such structures in the course of the present study. During drug-induced death of HeLa cells after mitotic block, internucleosomal fragmentation of DNA has been detected in some circumstances (Jordon et al, 1996) and not in others (Lock and Stribinskiene, 1996). DNA ladders were not evident during etoposide-induced death of murine fibroblasts after $G_2$/M arrest (Bonelli et al, 1996). More relevantly, absence of ladders was noted in L1210 leukaemic cells after cisplatin-induced $G_2$/M arrest (Ormerod et al, 1994). The present findings include detection of DNA ladders 48 h after the addition of 0.5 μM etoposide to CEM cells but not in MOLT-4 cells (Figure 6), although all our other findings suggest the mode of etoposide-induced cell death in these two populations is the same. In fact, the diverse observations summarized above provide no basis for establishing a relationship between $G_2$/M arrest and cell death. However, at least in respect of the present data, a relationship may be proposed by reference to the PFGE results.

Reviewing the description of higher-order fragmentation during apoptosis in many contexts, Cidlowski and colleagues (Bortner et al, 1995) commented that more studies were needed to determine whether higher-order fragmentation is indeed a universal

© Cancer Research Campaign 1998

characteristic of apoptosis. As MOLT-4 cells were significant in demonstrating the limitations of DNA ladders as a molecular indicator of apoptosis (Hotz et al, 1992; Catchpoole and Stewart, 1993; Falcieri et al, 1993), detection of higher-order fragmentation in these cells after exposure to etoposide is noteworthy. Such a result was reported by Beere et al (1995) using 50 μM etoposide. It is now evident that concentrations of the drug 1000-fold less may induce the same effect in these cells, albeit after a markedly different exposure time (Figure 5). Our studies indicate no difference in sensitivity for 50-kb breakage between MOLT-4 and CEM cells. The two lines are equally sensitive to the drug [(Catchpoole and Stewart, 1993) and Figure 1] and this is consistent with virtually identical dose–response patterns for 50-kb fragmentation.

After the exposure of CEM or MOLT-4 cells to 5 μM etoposide, the appearance of 50 kb fragments (Table 2) is virtually coincident with topoisomerase II-mediated single-strand breakage, production of DNA ladders and histological evidence of cell death (Catchpoole and Stewart, 1993). In each of the lines exposed to either 0.5 μM or 0.05 μM etoposide, higher-order DNA fragmentation was detectable by PFGE after 12 h, although there was no morphological evidence of cell death at that time (Table 1, Figure 2). Under these conditions, 50-kb breakage coincided with $G_2$/M arrest. The inability to detect ladders 24 h after the exposure of CEM cells to 0.5 μM etoposide was a consistent finding. However, 48 h after the addition of 0.5 μM etoposide, loss of membrane integrity was clearly evident in both lines (Figure 7) and DNA ladders were evident in CEM cells (Figure 6). The separate genesis of 50 kb and internucleosomal fragments, respectively, is compatible with other reports of higher-order fragmentation in the absence of DNA ladders (Oberhammer et al, 1993; Watanabe et al, 1995) and evidence that, in thymocytes, nuclease activity mediating cleavage of DNA into large fragments is distinguished from that causing ladders (Sun and Cohen, 1994; Walker et al, 1994).

It is evident from the present findings that DNA ladders are a limited, if not an inadequate vehicle to establish a specific mode of cell death. As exemplified by MOLT-4 cells, ladders are not observed when all other criteria of apoptosis, including 50-kb breakage, are apparent. More critically, as exemplified by CEM cells, ladders are an insensitive analytical tool that are observed in a narrow range, both in terms of time and drug concentration, compared with higher-order fragmentation. The remarkably restricted dose/time distribution of ladders (L) in Table 6 suggests that at least some reports concerning absence of this lesion in death after $G_2$/M arrest involve missing the narrow 'window' at which DNA ladders appear. Finally, in the experimental system described here, DNA ladders are coincident with, and do not precede membrane and morphological evidence of cell death, confirming their status as a post-mortem event (Vaux and Strasser, 1996). None of these limitations apply to 50-kb fragmentation of DNA.

Higher-order fragmentation of DNA, and specifically generation of 50-kb breaks, has been associated with apoptotic cell death. The present results using 5 μM etoposide provide further evidence for such an association in the case of CEM cells and, in the case of MOLT-4 cells, indicate that such breakage may occur more generally than another indicator, namely DNA ladders. Processes associated with drug-induced apotosis have been characterized in systems exemplified by treatment of these two lines with 5 μM etoposide: cell death occurs in 4–8 h. Drug-induced cell death may also occur over a wider timeframe, and specifically after $G_2$/M arrest. An argument has been made that such cell death occurs by processes that distinguish it from apoptosis. Our findings mitigate caution in this regard. First, apoptotic indicators (typified by DNA ladders in this study) may be not so much absent as difficult to detect in a broader timeframe. Second, certain indicators (commitment to differentiation and 50-kb DNA breakage) were observed by us to be common to apoptotic cell death and to cell death after $G_2$/M arrest. Moreover, in the latter circumstances, 50-kb DNA breakage clearly precedes both DNA ladders and morphological change by light microscopy. Finally, in etoposide-treated CEM and MOLT-4 cells, 50-kb DNA breakage is best characterized as being coincident with $G_2$/M arrest rather than a consequence of it. This mode of cell death seems closely related to apoptosis, but may be particular to haematopoietic cells.

### ACKNOWLEDGEMENTS

This work was supported by grants from the National Health and Medical Research Council (Australia) and the New South Wales Cancer Council. The Children's Cancer Research Institute is supported by the Children's Cancer Institute Australia.

### REFERENCES

Allen PD, Bustin SA and Newland AC (1993) The role of apoptosis (programmed cell death) in hemopoiesis and the immune system. *Blood* **7**: 63–73

An B and Dou QP (1996) Cleavage of retinoblastoma protein during apoptosis: An interleukin 1β-converting enzyme-like protease as candidate. *Cancer Res* **56**: 438–442

Barres BA, Hart IK, Coles HSR, Burne JF, Voyvodic JT, Richardson WD and Raff MC (1992) Cell death and control of cell survival in the oligodendrocyte lineage. *Cell* **70**: 31–46

Barry MA and Eastman A (1992) Endonuclease activation during apoptosis: The role of cytosolic $Ca^{2+}$ and pH. *Biochem Biophys Res Commun* **186**: 782–789

Barry MA, Behnke CA and Eastman A (1990) Activition of programmed cell death (apoptosis) by cisplatin, other anticancer drugs, toxins and hyperthermia. *Biochem Pharmacol* **40**: 2353–2362

Beere HM, Chresta CM, Alejo-Hergerg A, Skladanowski A, Dive C, Larsen AK and Hickman JA (1995) Investigation of the mechanism of higher order chromatin fragmentation observed in drug-induced apoptosis. *Mol Pharmacol* **47**: 986–996

Bertrand R, Sarange M, Jenkin J, Kerrigan D and Pommier Y (1991) Differential induction of secondary DNA fragmentation by topoisomerase II inhibitors in human tumor cell lines with amplified *c-myc* expression. *Cancer Res* **51**: 6280–6285

Bonelli G, Sacchi MC, Barbiero G, Duranti F, Goglio G, Verdun di Cantogno L, Amenta JS, Piacentini M, Tacchetti C and Baccino FM (1996) Apoptosis of L929 cells by etoposide: A quantitative and kinetic approach. *Exp Cell Res* **228**: 292–305

Bordow SB, Haber M, Madafiglio J, Cheung B, Marshall GM and Norris MD (1994) Expression of the multidrug resistance-associated protein (MRP) is correlated with amplification and over-expression of the N-myc oncogene in primary human neuroblastoma and cell lines. *Cancer Res* **54**: 5036–5040

Bortner CD, Oldenburg NBE and Cidlowski JA (1995) The role of DNA fragmentation in apoptosis. *Trends Cell Biol* **5**: 21–26

Brown DG, Sun X-M and Cohen GM (1993) Dexamethasone-induced apoptosis involves cleavage of DNA to large fragments prior to internucleosomal fragmentation. *J Biol Chem* **268**: 3037–3039

Campana D and Janossy G (1988) Proliferation of normal and malignant human immature lymphoid cells. *Blood* **71**: 1201–1210

Catchpoole DR and Stewart BW (1993) Etoposide-induced cytotoxicity in two human T-cell leukemic lines: Delayed loss of membrane permeability rather than DNA fragmentation as an indicator of programmed cell death. *Cancer Res* **53**: 4287–4296

Catchpoole DR and Stewart BW (1995) Formation of apoptotic bodies is associated with internucleosomal DNA fragmentation during drug-induced apoptosis. *Exp Cell Res* **215**: 169–177

Chang WP and Little JB (1991) Delayed reproductive death in X-irradiated Chinese hamster ovary cells. *Int J Radiat Biol* **60**: 483–496

© Cancer Research Campaign 1998

Chen I-T, Smith ML, O'Connor PM and Fornace AJJr (1995) Direct interaction of Gadd45 with PCNA and evidence for competitive interaction of Gadd45 and p21$^{Waf1/Cip1}$ with PCNA. *Oncogene* **11**: 1931–1937

Chomczynski P and Sacchi N (1987) Single step method of RNA isolation by guanidium thiocyanate-phenol-chloroform extraction. *Anal Biochem* **162**: 156–159

Chow K-C and Ross WE (1987) Topoisomerase-specific drug sensitivity in relation to cell cycle progression. *Mol Cell Biol* **7**: 3119–3123

Cohen GM, Sun XM, Fearnhead H, MacFarlane M, Brown DG, Snowden RT and Dinsdale D (1994) Formation of large molecular weight fragments of DNA is a key committed step of apoptosis in thymocytes. *J Immunol* **153**: 507–516

Compton MM (1992) A biological hallmark of apoptosis: Internucleosomal degradation of the genome. *Cancer Metastas Rev* **11**: 105–119

Constantinou A, Grdina D, Kiguchi K and Huberman E (1992) The effect of topoisomerase inhibitors on the expression of differentiation markers and cell cycle progression in human K562 leukemia cells. *Exp Cell Res* **203**: 100–106

da Silva CP, de Oliveira CR and de Lima AP (1996) Apoptosis as a mechanism of cell death induced by different chemotherapeutic drugs in human leukaemic T-lymphocytes. *Biochem Pharmocol* **51**: 1331–1340

Deitch AD, Law H and White RD (1982) A stable propidium iodide staining procedure for flow cytometry. *J Histochem Cytochem* **30**: 967–972

Del Bino G, Skierski JS and Darzynkiewicz Z (1991) The concentration-dependent diversity of effects of DNA topoisomerase I and II inhibitors on the cell cycle of HL-60 cells. *Exp Cell Res* **195**: 485–491

Demarcq C, Bunch RT, Creswell D and Eastman A (1994) The role of cell cycle progression in cisplatin-induced apoptosis in Chinese hamster ovary cells. *Cell Growth Diff* **5**: 983–993

Desjardins LM and MacManus JP (1995) An adherent cell model to study different stages of apoptosis. *Exp Cell Res* **216**: 380–387

Dou QP, An B and Will PL (1995) Induction of a retinoblastoma phosphatase activity by anticancer drugs accompanies p53-independent $G_1$ arrest and apoptosis. *Proc Natl Acad Sci USA* **92**: 9019–9023

Eliopoulos AG, Kerr DJ, Herod J, Hodgkins L, Krajewski S, Reed JC and Young LS (1995) The control of apoptosis and drug resistance in ovarian cancer: Influence of p53 and bcl-2. *Oncogene* **11**: 1217–1228

Falcieri E, Martelli AM, Bareggi R, Cataldi A and Cocco L (1993) The protein kinase inhibitor staurosporine induces morphological changes typical of apoptosis in MOLT-4 cells without concomitant DNA fragmentation. *Biochem Biophys Res Commun* **193**: 19–25

Filipski I, Leblanc J, Youdale T, Sikorska M and Walker PR (1990) Periodicity of DNA folding in higher order chromatin structures. *EMBO J* **9**: 1319–1327

Grant S, Freemerman AJ, Birrer MJ, Martin HA, Turner AJ, Szabo E, Chelliah J and Jarvis WD (1996) Effect of 1-β-D-arabinofuranosylcytosine on apoptosis and differentiation on human monocytic leukaemia cells (U937) expressing a c-jun dominant-negative mutant protein (TAM67). *Cell Growth Diff* **7**: 603

Hickman JA (1992) Apoptosis induced by anticancer drugs. *Cancer Metast Rev* **11**: 121–139

Hotz MA, Del Bino G, Lassota P, Traganos F and Darzynkiewcz Z (1992) Cytostatic and cytotoxic effects of fostriecin on human promyelocytic HL-60 and lymphocytic MOLT-4 leukemic cells. *Cancer Res* **52**: 1530–1535

Jordon MA, Wendell K, Gardiner S, Derry WB, Copp H and Wilson L (1996) Mitotic block induced in HeLa cells by low concentrations of paclitaxel (taxol) results in abnormal mitotic exit and apoptotic cell death. *Cancer Res* **56**: 816–825

Lazebnik YA, Takahashi A, Moir RD, Goldman RD, Poirier GG, Kaufmann SH and Earnshaw WC (1995) Studies of the lamin proteinase reveal multiple parallel biochemical pathways during apoptotic execution. *Proc Natl Acad Sci USA* **92**: 9042–9046

Lennon SV, Martin SJ and Cotter TG (1991) Dose-dependent induction of apoptosis in human tumour cell lines by widely diverging stimuli. *Cell Prolif* **24**: 203–214

Li CJ, Wang C and Pardee AB (1995) Induction of apoptosis by β-lapachone in human prostate cancer cells. *Cancer Res* **55**: 3712–3715

Lock RB and Stribinskiene L (1996) Dual modes of death induced by etoposide in human epithelial tumour cells allow bcl-2 to inhibit apoptosis without affecting clonogenic survival. *Cancer Res* **56**: 4006–4012

Lock RB, Galperina OV, Feldhoff RC and Rhodes LJ (1994) Concentration-dependent differences in the mechanisms by which caffeine potentiates etoposide cytotoxicity in HeLa cells. *Cancer Res* **54**: 4933–4939

Lowe SW, Schmitt EM, Smith SW, Osborne BA and Jacks T (1993) p53 is required for radiation-induced apoptosis in mouse thymocytes. *Nature* **362**: 847–849

Lowe SW, Bodis S, McClatchey A, Remington L, Ruley HE, Fisher DE, Housman DE and Jacks T (1994) P53 status and the efficacy of cancer therapy in vivo. *Science* **266**: 807–810

Ma DDF, Sylwestrowicz T, Janossy G and Hoffbrand AV (1983) The role of purine metabolic enzymes and terminal deoxynucleotidyl tranferase in intrathymic T-cell differentiation. *Immunol Today* **4**: 65–68

Madrid-Marina V, Martinez-Valdez H and Cohen A (1990) Phorbol esters induce changes in adenosine deaminase, purine nucleoside phosphorylase, and terminal deoxynucleotidyl transferase messenger RNA levels in human leukemic cell lines. *Cancer Res* **50**: 2891–2894

Marks DI and Fox RM (1991) DNA damage, poly (ADP-ribosyl)ation and apoptotic cell death as a potential common pathway of cytotoxic drug action. *Biochem Pharmocol* **42**: 1859–1867

Marks DI, Kurz BW, Link MP, Ng E, Shuster JJ, Lauer SJ, Brodsky I and Haines DS (1996) High incidence of potential p53 inactivation in poor outcome childhood acute lymphoblastic leukaemia at diagnosis. *Blood* **87**: 1155–1161

Martinez-Valdez H and Cohen A (1988) Coordinate regulation of mRNAs encoding adenosine deaminase, purine nucleoside phosphorylase, and terminal deoxynucleotidyltransferase by phorbol esters in human thymocytes. *Proc Natl Acad Sci USA* **85**: 6900–6903

Mross KB (1996) The pharmacokinetics of epipodopyllotoxins – clinical relevance. In *Acute Leukemias V: Experimental Approaches and Management of Refractory Disease*. Hiddemann W, Buchner T, Wormann B, Ritter J, Creutzig U, Plunkett W and Keating M (eds), pp. 54–61. Springer: Berlin

Noonan KE, Beck C, Holzmayer TA, Chin JE, Wunder JS, Andrulis IL, Gazdar AF, Willman CL, Griffith B, Von Hoff DD and Roninson IB (1990) Quantitative analysis of MDR1 (multidrug resistance) gene expression in human tumors by polymerase chain reaction. *Proc Natl Acad Sci USA* **87**: 7160–7164

Oberhammer F, Wilson JW, Dive C, Morris ID, Hickman JA, Wakeling AE, Walker PR and Sikorska M (1993) Apoptotic death in epithelial cells: Cleavage of DNA to 300 and or 50 kb fragments prior to or in the absence of internucleosomal fragmentation. *EMBO J* **12**: 3679–3684

Oberhammer FA, Pavelka M, Sharma S, Tiefenbacher R, Purchio AF, Bursch W and Schulte-Hermann R (1922) Induction of apoptosis in cultured hepatocytes and in regressing liver by transforming growth factor β1. *Proc Natl Acad Sci USA* **89**: 5408–5412

Ohta H, Sweeney EA, Masamune A, Yatomi Y, Hakomori S and Igarashi Y (1995) Induction of apoptosis by sphingosine in human-leukemic HL-60 cells: A possible endogenous modulator of apoptosis DNA fragmentation occurring during phorbol ester-induced differentiation. *Cancer Res* **55**: 691–697

Ormerod MG, Orr RM and Peacock JH (1994) The role of apoptosis in cell killing by cisplatin: A flow cytometric study. *Br J Cancer* **69**: 93–100

Rao AP and Rao PN (1976) The cause of $G_2$-arrest in Chinese hamster ovary cells treated with anticancer drugs. *J Natl Cancer Inst* **57**: 1139–1143

Rusch V, Klimstra D, Venkatraman E, Oliver J, Martini N, Gralla R, Kris M and Dmitrovsky E (1995) Aberrant p53 expression predicts clinical resistance to cisplatin-based chemotherapy in locally advanced non-small cell lung cancer. *Cancer Res* **55**: 5038–5042

Sachs L and Lotem J (1993) Control of programmed cell death in normal and leukemic cells: new implications for therapy. *Blood* **82**: 15–21

Salmon M, Pilling D, Borthwick NJ, Viner N, Janossy G, Bacon PA and Akbar AN (1994) The progressive differentiation of primed T cells is associated with an increasing susceptibility to apoptosis. *Eur J Immunol* **24**: 892–899

Schimke RT, Kung A, Sherwood SS, Sheridan J and Sharma R (1995) Life, death and genomic change in perturbed cell cycles. In *The Role of Apoptosis in Development, Tissue Homeostasis and Malignancy: Death from Inside Out*. Dexter TM, Raff MC and Wyllie AH (eds), pp. 75–81. Chapman & Hall: London

Schwartz PM, Barnett SK, Atillasoy ES and Milstone LM (1992) Methotrexate induces differentiation of human keratinocytes. *Proc Natl Acad Sci USA* **89**: 594–598

Smith GK, Duch DS, Dev IK and Kaufmann SH (1992) Metabolic effects and kill of human T-cell leukemia by 5-deazaacyclotetrahydrofolate, a specific inhibitor of glycineamide ribonucleotide transformylase. *Cancer Res* **52**: 4895–4903

Sorenson CM, Barry MA and Eastman A (1990) Analysis of events associated with cell cycle arrest at G2 phase and cell death induced by cisplatin. *J Natl Cancer Inst* **82**: 749–755

Stewart BW (1994) Mechanisms of apoptosis: Integration of genetic, biochemical and cellular indicators. *J Natl Cancer Inst* **86**: 1286–1296

Stewart BW, Sleiman RJ and Catchpoole DR (1995) Relationships between cell cycle distribution, differentiation and apoptosis in etoposide-treated human leukemic cells (abstract). *Proc Am Assoc Cancer Res* **36**: 19

Sun X-M and Cohen GM (1994) $Mg^{2+}$-dependent cleavage of DNA into kilobase pair fragments is responsible for the initial degradation of DNA in apoptosis. *J Biol Chem* **269**: 14857–14860

© Cancer Research Campaign 1998

Tornaletti S and Pfeifer GP (1995) Complete and tissue-independent methylation of CpG sites in the p53 gene: Implications for mutations in human cancer. *Oncogene* **10**: 1493–1499

Touneki O, Pron G, Belehradek JJr and Mir LM (1993) Bleomycin, an apoptosis-mimetic drug that induces two types of cell death depending on the number of molecules internalized. *Cancer Res* **53**: 5462–5469

Vaux DL and Strasser A (1996) The molecular biology of apoptosis. *Proc Natl Acad Sci USA* **93**: 2239–2244

Walker PR, Weaver VM, Lach B, Leblanc J and Sikorska M (1994) Endonuclease activities associated with high molecular weight and internucleosomal DNA fragmentation in apoptosis. *Exp Cell Res* **213**: 100–106

Warrell RP (1997) Differiation Agents. In *Cancer: Principles and Practice of Oncology*, 5th edn, DeVita VT, Hellman S and Rosenberg SA (eds), pp. 483–490. Lippincott-Raven: Philadelphia

Watanabe H, Kanbe K, Shinozaki T, Hoshino H and Chigira M (1995) Apoptosis of a fibrosarcoma induced by protein-free culture involves DNA cleavage to large fragments but not internucleosomal fragmentation. *Int J Cancer* **62**: 191–198

Weis M, Schlegel J, Kass GEN, Holmstrom TH, Peters I, Eriksson J, Orrenius S and Chow SC (1995) Cellular events in Fas/APO-1-mediated apoptosis in JURKAT T lymphocytes. *Exp Cell Res* **219**: 699–708

Wyllie AH (1987) Apoptosis: Cell death in tissue regulation. *J Pathol* **153**: 313–316

Zhivotovsky B, Wade D, Gahm A, Orrenius S and Nicotera P (1994) Formation of 50 kbp chromatin fragments in isolated liver nuclei is mediated by protease and endonuclease activation. *FEBS Lett* **351**: 150–154

© Cancer Research Campaign 1998


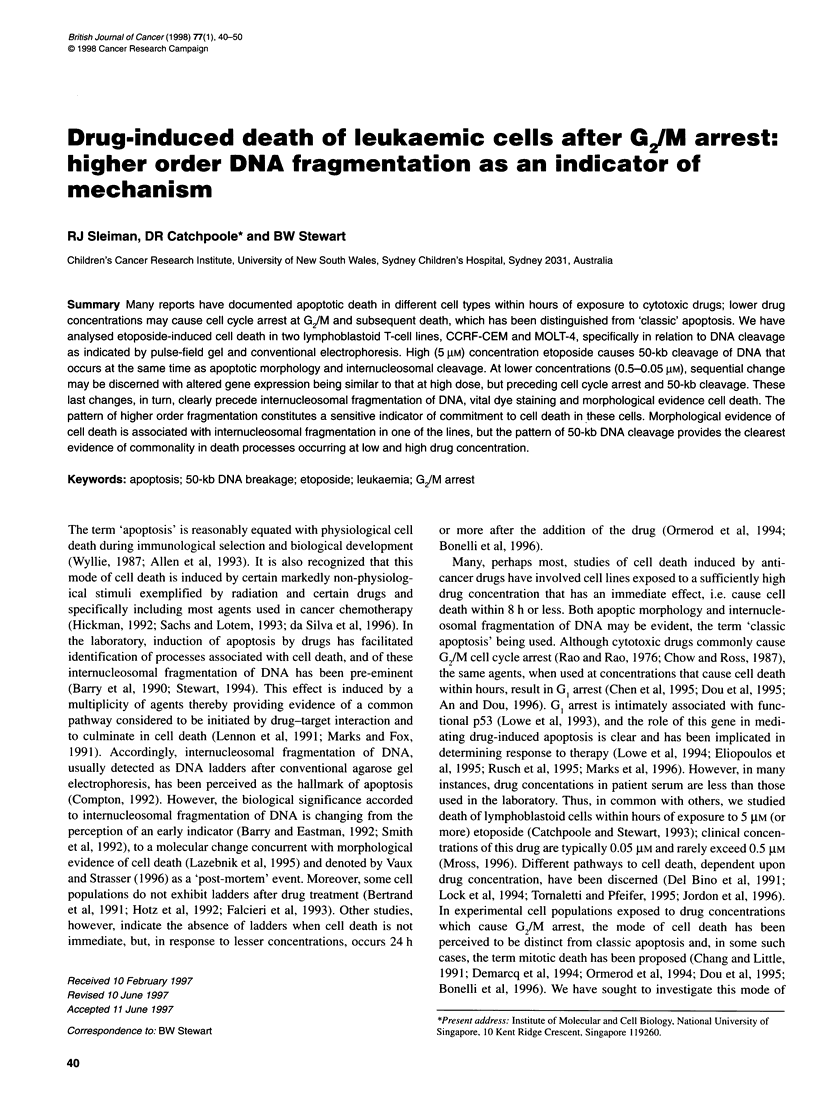

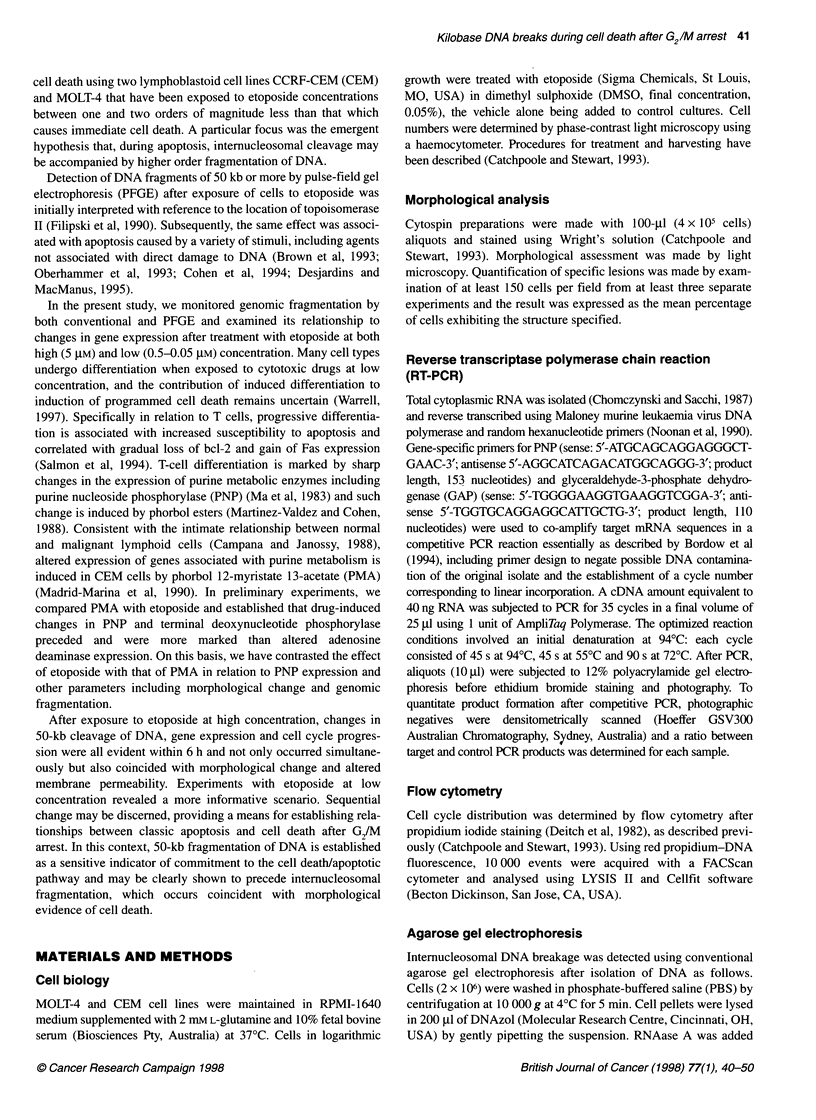

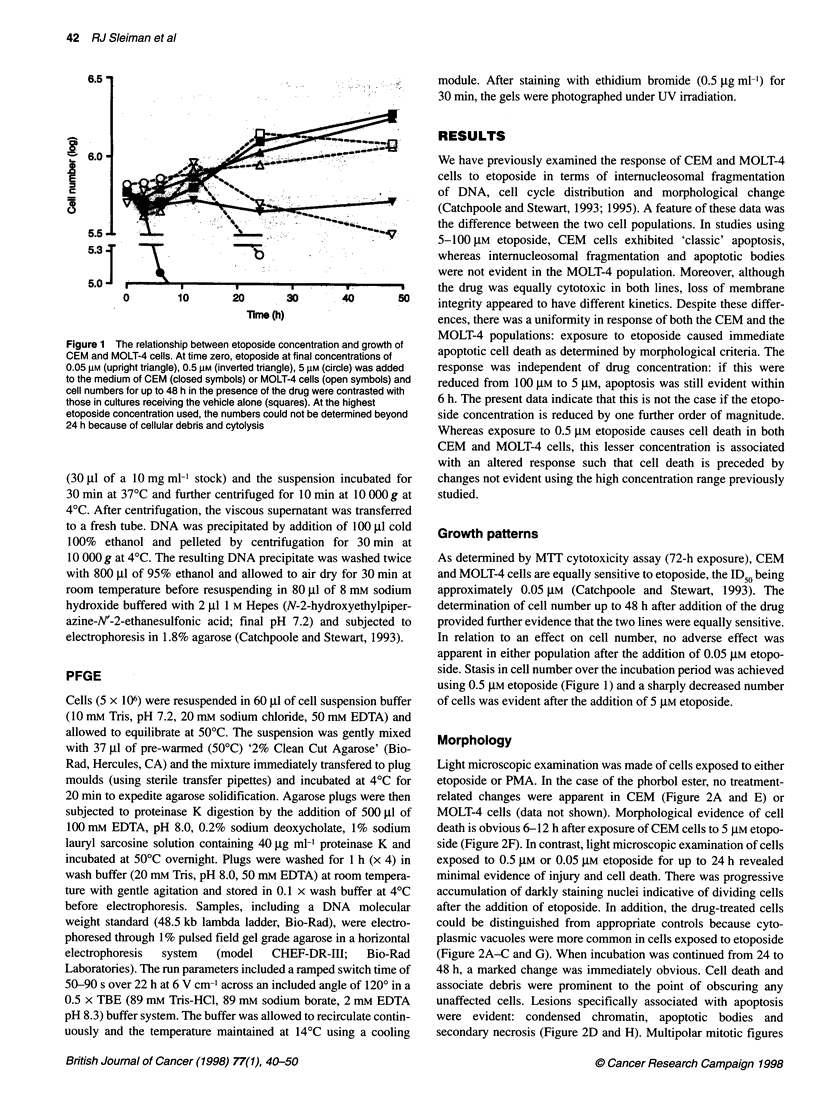

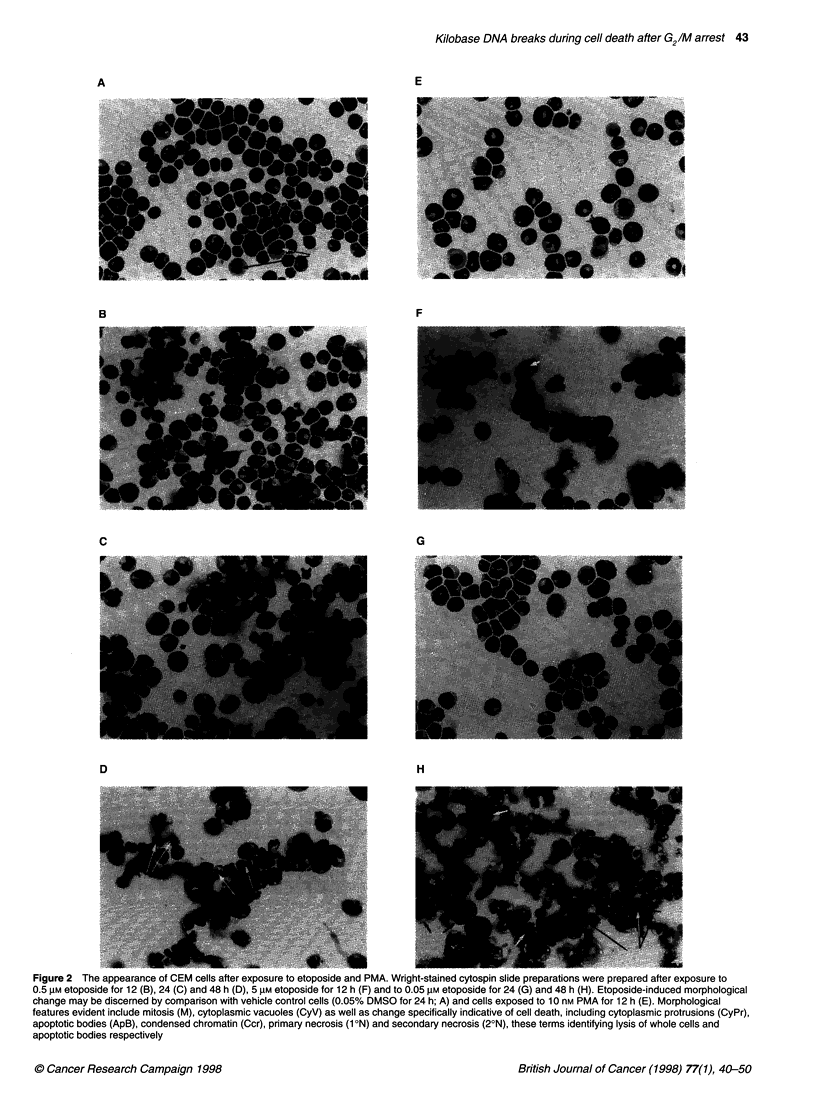

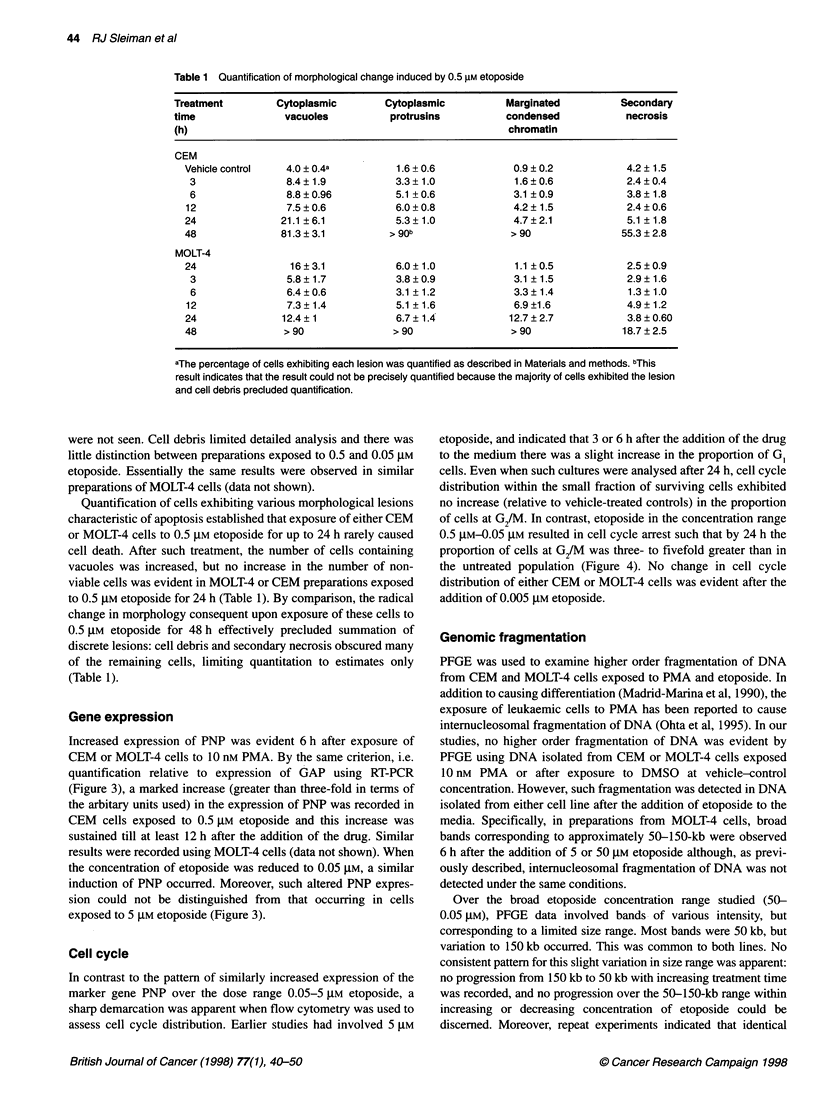

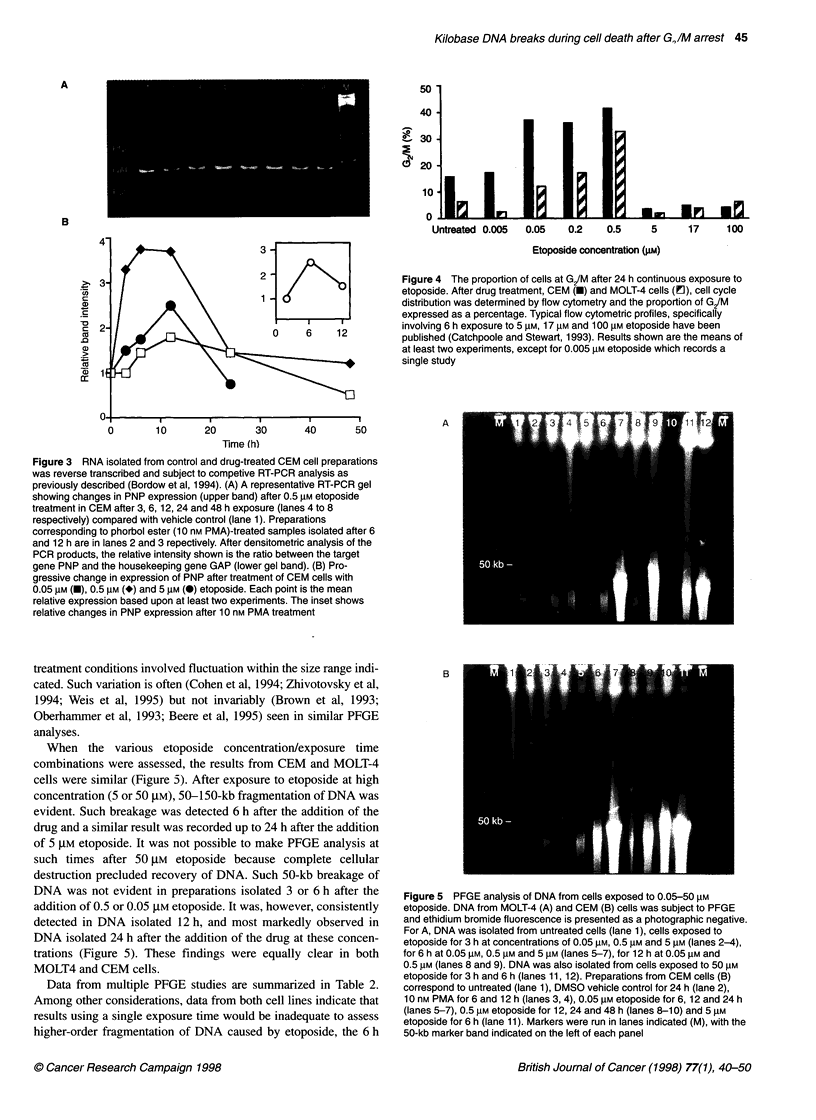

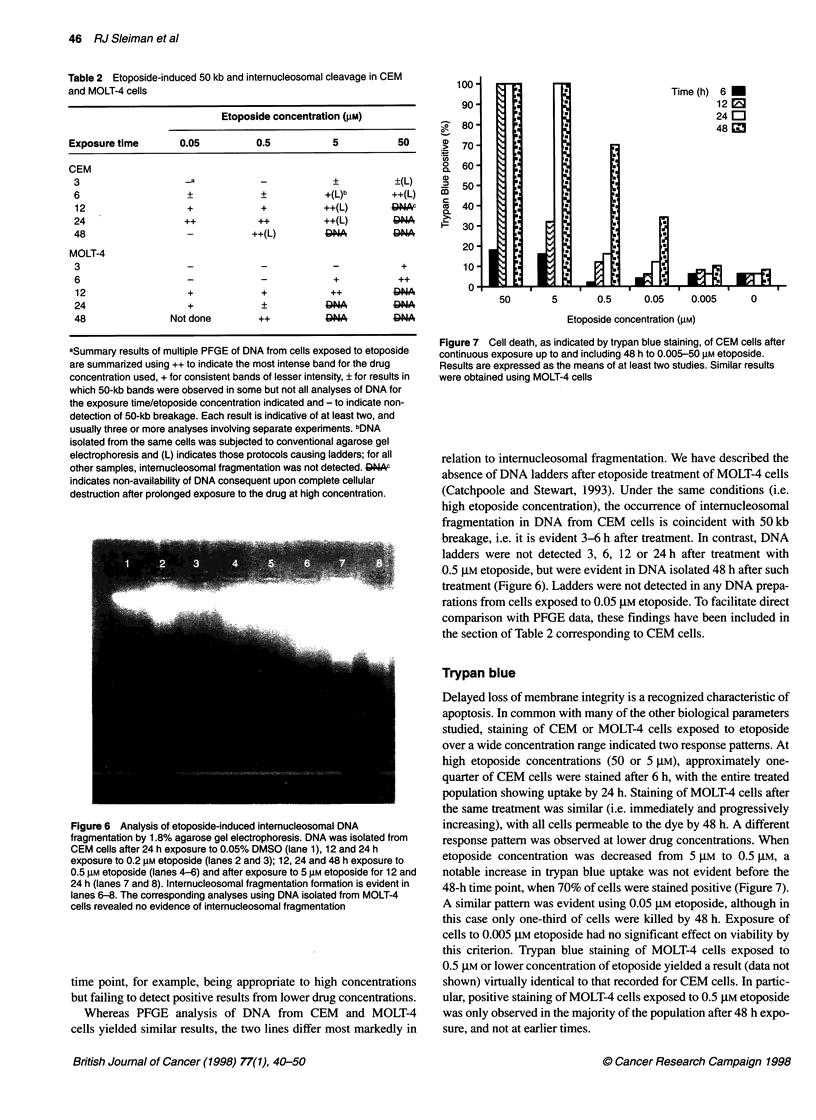

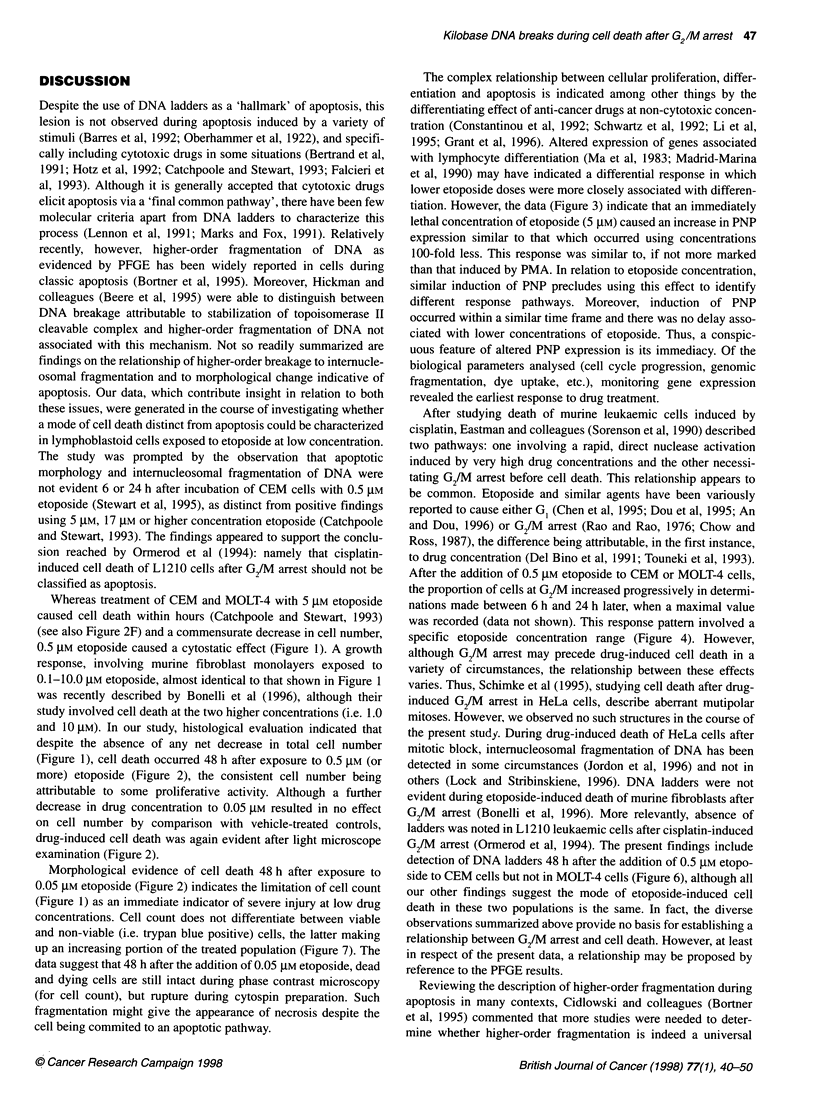

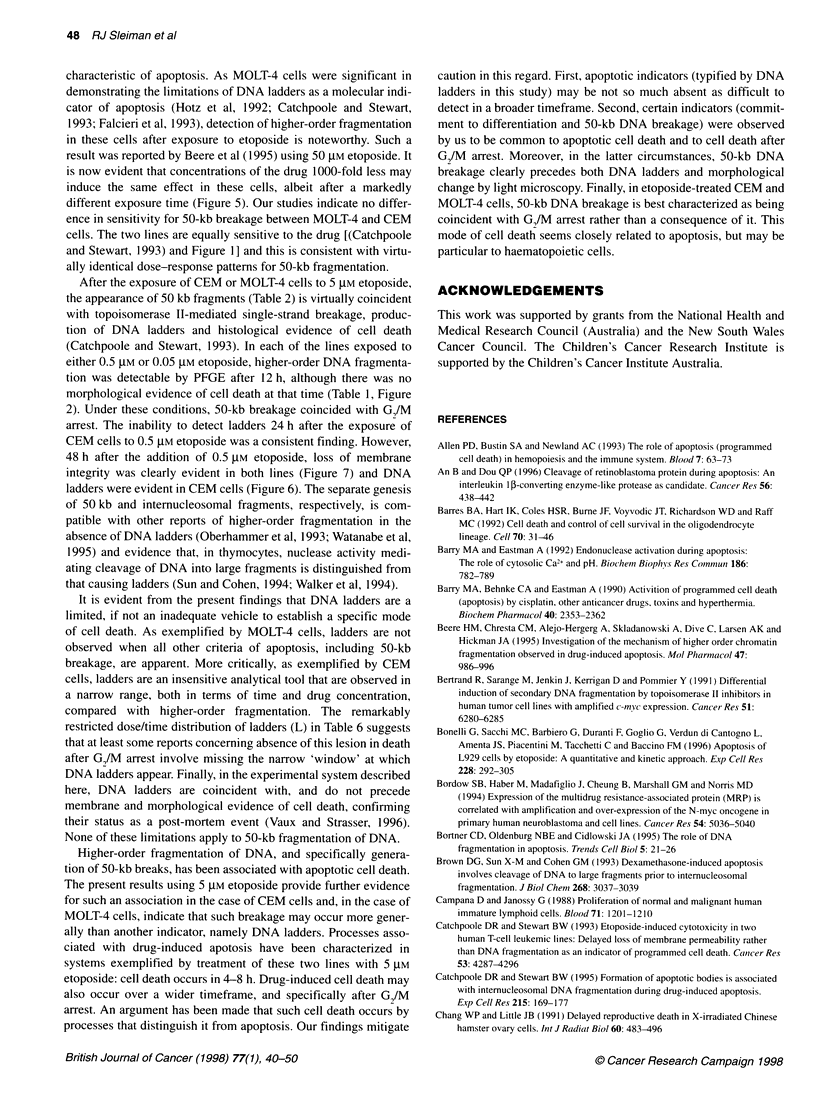

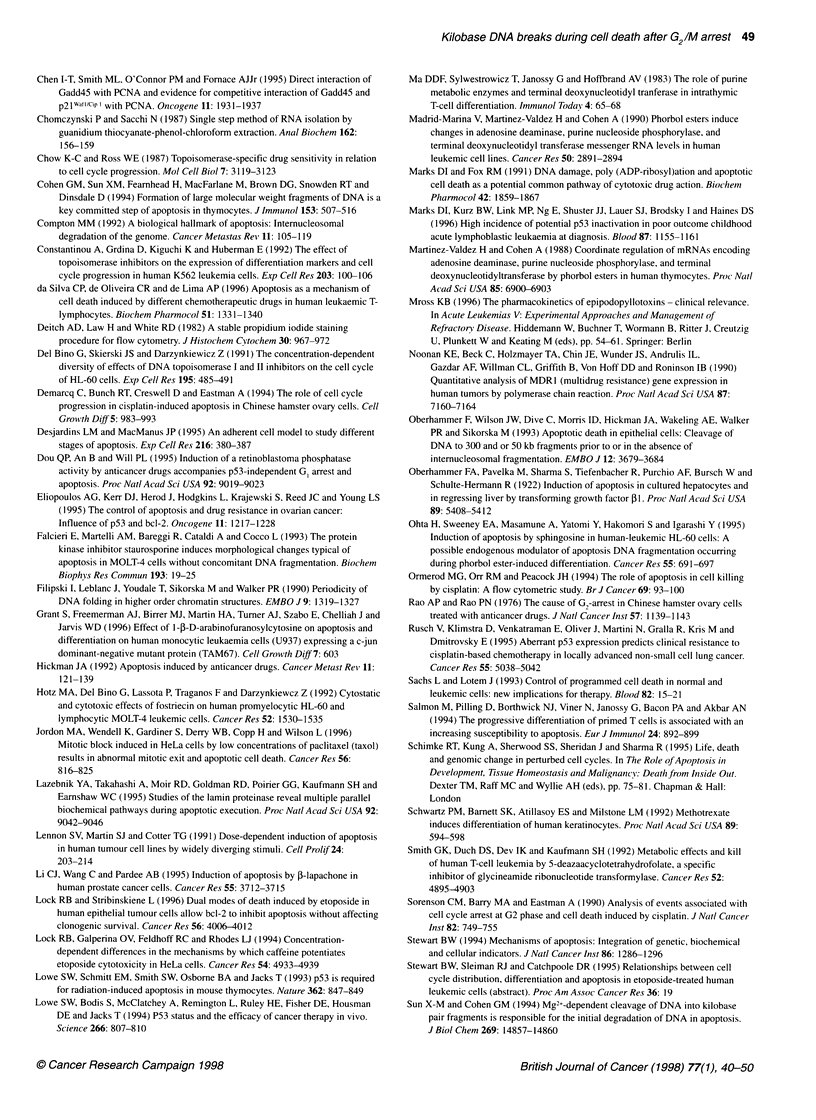

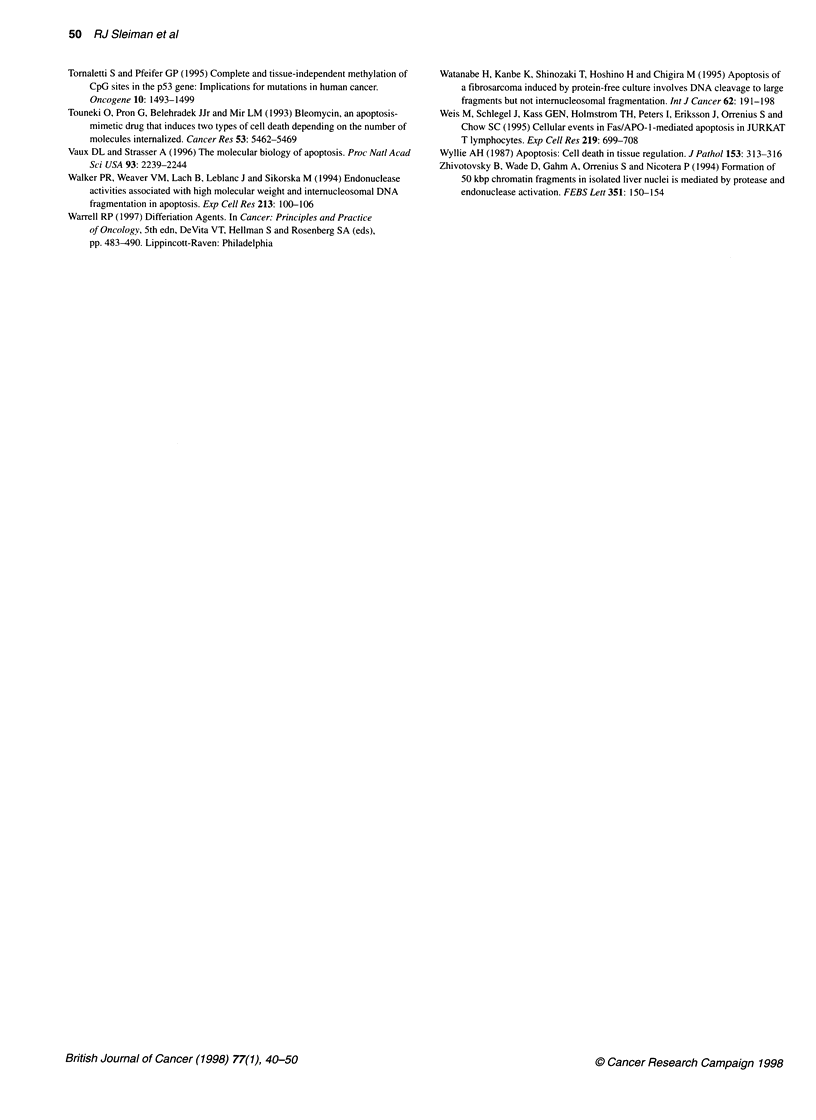


## References

[OCR_00983] Allen P. D., Bustin S. A., Newland A. C. (1993). The role of apoptosis (programmed cell death) in haemopoiesis and the immune system.. Blood Rev.

[OCR_00987] An B., Dou Q. P. (1996). Cleavage of retinoblastoma protein during apoptosis: an interleukin 1 beta-converting enzyme-like protease as candidate.. Cancer Res.

[OCR_00992] Barres B. A., Hart I. K., Coles H. S., Burne J. F., Voyvodic J. T., Richardson W. D., Raff M. C. (1992). Cell death and control of cell survival in the oligodendrocyte lineage.. Cell.

[OCR_01002] Barry M. A., Behnke C. A., Eastman A. (1990). Activation of programmed cell death (apoptosis) by cisplatin, other anticancer drugs, toxins and hyperthermia.. Biochem Pharmacol.

[OCR_00997] Barry M. A., Eastman A. (1992). Endonuclease activation during apoptosis: the role of cytosolic Ca2+ and pH.. Biochem Biophys Res Commun.

[OCR_01007] Beere H. M., Chresta C. M., Alejo-Herberg A., Skladanowski A., Dive C., Larsen A. K., Hickman J. A. (1995). Investigation of the mechanism of higher order chromatin fragmentation observed in drug-induced apoptosis.. Mol Pharmacol.

[OCR_01013] Bertrand R., Sarang M., Jenkin J., Kerrigan D., Pommier Y. (1991). Differential induction of secondary DNA fragmentation by topoisomerase II inhibitors in human tumor cell lines with amplified c-myc expression.. Cancer Res.

[OCR_01021] Bonelli G., Sacchi M. C., Barbiero G., Duranti F., Goglio G., Verdun di Cantogno L., Amenta J. S., Piacentini M., Tacchetti C., Baccino F. M. (1996). Apoptosis of L929 cells by etoposide: a quantitative and kinetic approach.. Exp Cell Res.

[OCR_01025] Bordow S. B., Haber M., Madafiglio J., Cheung B., Marshall G. M., Norris M. D. (1994). Expression of the multidrug resistance-associated protein (MRP) gene correlates with amplification and overexpression of the N-myc oncogene in childhood neuroblastoma.. Cancer Res.

[OCR_01031] Bortner C. D., Oldenburg N. B., Cidlowski J. A. (1995). The role of DNA fragmentation in apoptosis.. Trends Cell Biol.

[OCR_01035] Brown D. G., Sun X. M., Cohen G. M. (1993). Dexamethasone-induced apoptosis involves cleavage of DNA to large fragments prior to internucleosomal fragmentation.. J Biol Chem.

[OCR_01040] Campana D., Janossy G. (1988). Proliferation of normal and malignant human immature lymphoid cells.. Blood.

[OCR_01044] Catchpoole D. R., Stewart B. W. (1993). Etoposide-induced cytotoxicity in two human T-cell leukemic lines: delayed loss of membrane permeability rather than DNA fragmentation as an indicator of programmed cell death.. Cancer Res.

[OCR_01051] Catchpoole D. R., Stewart B. W. (1995). Formation of apoptotic bodies is associated with internucleosomal DNA fragmentation during drug-induced apoptosis.. Exp Cell Res.

[OCR_01056] Chang W. P., Little J. B. (1991). Delayed reproductive death in X-irradiated Chinese hamster ovary cells.. Int J Radiat Biol.

[OCR_01064] Chen I. T., Smith M. L., O'Connor P. M., Fornace A. J. (1995). Direct interaction of Gadd45 with PCNA and evidence for competitive interaction of Gadd45 and p21Waf1/Cip1 with PCNA.. Oncogene.

[OCR_01069] Chomczynski P., Sacchi N. (1987). Single-step method of RNA isolation by acid guanidinium thiocyanate-phenol-chloroform extraction.. Anal Biochem.

[OCR_01074] Chow K. C., Ross W. E. (1987). Topoisomerase-specific drug sensitivity in relation to cell cycle progression.. Mol Cell Biol.

[OCR_01078] Cohen G. M., Sun X. M., Fearnhead H., MacFarlane M., Brown D. G., Snowden R. T., Dinsdale D. (1994). Formation of large molecular weight fragments of DNA is a key committed step of apoptosis in thymocytes.. J Immunol.

[OCR_01082] Compton M. M. (1992). A biochemical hallmark of apoptosis: internucleosomal degradation of the genome.. Cancer Metastasis Rev.

[OCR_01086] Constantinou A., Grdina D., Kiguchi K., Huberman E. (1992). The effect of topoisomerase inhibitors on the expression of differentiation markers and cell cycle progression in human K-562 leukemia cells.. Exp Cell Res.

[OCR_01095] Deitch A. D., Law H., deVere White R. (1982). A stable propidium iodide staining procedure for flow cytometry.. J Histochem Cytochem.

[OCR_01099] Del Bino G., Skierski J. S., Darzynkiewicz Z. (1991). The concentration-dependent diversity of effects of DNA topoisomerase I and II inhibitors on the cell cycle of HL-60 cells.. Exp Cell Res.

[OCR_01104] Demarcq C., Bunch R. T., Creswell D., Eastman A. (1994). The role of cell cycle progression in cisplatin-induced apoptosis in Chinese hamster ovary cells.. Cell Growth Differ.

[OCR_01109] Desjardins L. M., MacManus J. P. (1995). An adherent cell model to study different stages of apoptosis.. Exp Cell Res.

[OCR_01113] Dou Q. P., An B., Will P. L. (1995). Induction of a retinoblastoma phosphatase activity by anticancer drugs accompanies p53-independent G1 arrest and apoptosis.. Proc Natl Acad Sci U S A.

[OCR_01118] Eliopoulos A. G., Kerr D. J., Herod J., Hodgkins L., Krajewski S., Reed J. C., Young L. S. (1995). The control of apoptosis and drug resistance in ovarian cancer: influence of p53 and Bcl-2.. Oncogene.

[OCR_01123] Falcieri E., Martelli A. M., Bareggi R., Cataldi A., Cocco L. (1993). The protein kinase inhibitor staurosporine induces morphological changes typical of apoptosis in MOLT-4 cells without concomitant DNA fragmentation.. Biochem Biophys Res Commun.

[OCR_01130] Filipski J., Leblanc J., Youdale T., Sikorska M., Walker P. R. (1990). Periodicity of DNA folding in higher order chromatin structures.. EMBO J.

[OCR_01134] Grant S., Freemerman A. J., Birrer M. J., Martin H. A., Turner A. J., Szabo E., Chelliah J., Jarvis W. D. (1996). Effect of 1-beta-D-arabinofuranosylcytosine on apoptosis and differentiation in human monocytic leukemia cells (U937) expressing a c-Jun dominant-negative mutant protein (TAM67).. Cell Growth Differ.

[OCR_01140] Hickman J. A. (1992). Apoptosis induced by anticancer drugs.. Cancer Metastasis Rev.

[OCR_01144] Hotz M. A., Del Bino G., Lassota P., Traganos F., Darzynkiewicz Z. (1992). Cytostatic and cytotoxic effects of fostriecin on human promyelocytic HL-60 and lymphocytic MOLT-4 leukemic cells.. Cancer Res.

[OCR_01149] Jordan M. A., Wendell K., Gardiner S., Derry W. B., Copp H., Wilson L. (1996). Mitotic block induced in HeLa cells by low concentrations of paclitaxel (Taxol) results in abnormal mitotic exit and apoptotic cell death.. Cancer Res.

[OCR_01155] Lazebnik Y. A., Takahashi A., Moir R. D., Goldman R. D., Poirier G. G., Kaufmann S. H., Earnshaw W. C. (1995). Studies of the lamin proteinase reveal multiple parallel biochemical pathways during apoptotic execution.. Proc Natl Acad Sci U S A.

[OCR_01162] Lennon S. V., Martin S. J., Cotter T. G. (1991). Dose-dependent induction of apoptosis in human tumour cell lines by widely diverging stimuli.. Cell Prolif.

[OCR_01167] Li C. J., Wang C., Pardee A. B. (1995). Induction of apoptosis by beta-lapachone in human prostate cancer cells.. Cancer Res.

[OCR_01176] Lock R. B., Galperina O. V., Feldhoff R. C., Rhodes L. J. (1994). Concentration-dependent differences in the mechanisms by which caffeine potentiates etoposide cytotoxicity in HeLa cells.. Cancer Res.

[OCR_01171] Lock R. B., Stribinskiene L. (1996). Dual modes of death induced by etoposide in human epithelial tumor cells allow Bcl-2 to inhibit apoptosis without affecting clonogenic survival.. Cancer Res.

[OCR_01185] Lowe S. W., Bodis S., McClatchey A., Remington L., Ruley H. E., Fisher D. E., Housman D. E., Jacks T. (1994). p53 status and the efficacy of cancer therapy in vivo.. Science.

[OCR_01181] Lowe S. W., Schmitt E. M., Smith S. W., Osborne B. A., Jacks T. (1993). p53 is required for radiation-induced apoptosis in mouse thymocytes.. Nature.

[OCR_01195] Madrid-Marina V., Martinez-Valdez H., Cohen A. (1990). Phorbol esters induce changes in adenosine deaminase, purine nucleoside phosphorylase, and terminal deoxynucleotidyl transferase messenger RNA levels in human leukemic cell lines.. Cancer Res.

[OCR_01201] Marks D. I., Fox R. M. (1991). DNA damage, poly (ADP-ribosyl)ation and apoptotic cell death as a potential common pathway of cytotoxic drug action.. Biochem Pharmacol.

[OCR_01206] Marks D. I., Kurz B. W., Link M. P., Ng E., Shuster J. J., Lauer S. J., Brodsky I., Haines D. S. (1996). High incidence of potential p53 inactivation in poor outcome childhood acute lymphoblastic leukemia at diagnosis.. Blood.

[OCR_01211] Martinez-Valdez H., Cohen A. (1988). Coordinate regulation of mRNAs encoding adenosine deaminase, purine nucleoside phosphorylase, and terminal deoxynucleotidyltransferase by phorbol esters in human thymocytes.. Proc Natl Acad Sci U S A.

[OCR_01225] Noonan K. E., Beck C., Holzmayer T. A., Chin J. E., Wunder J. S., Andrulis I. L., Gazdar A. F., Willman C. L., Griffith B., Von Hoff D. D. (1990). Quantitative analysis of MDR1 (multidrug resistance) gene expression in human tumors by polymerase chain reaction.. Proc Natl Acad Sci U S A.

[OCR_01238] Oberhammer F. A., Pavelka M., Sharma S., Tiefenbacher R., Purchio A. F., Bursch W., Schulte-Hermann R. (1992). Induction of apoptosis in cultured hepatocytes and in regressing liver by transforming growth factor beta 1.. Proc Natl Acad Sci U S A.

[OCR_01232] Oberhammer F., Wilson J. W., Dive C., Morris I. D., Hickman J. A., Wakeling A. E., Walker P. R., Sikorska M. (1993). Apoptotic death in epithelial cells: cleavage of DNA to 300 and/or 50 kb fragments prior to or in the absence of internucleosomal fragmentation.. EMBO J.

[OCR_01244] Ohta H., Sweeney E. A., Masamune A., Yatomi Y., Hakomori S., Igarashi Y. (1995). Induction of apoptosis by sphingosine in human leukemic HL-60 cells: a possible endogenous modulator of apoptotic DNA fragmentation occurring during phorbol ester-induced differentiation.. Cancer Res.

[OCR_01251] Ormerod M. G., Orr R. M., Peacock J. H. (1994). The role of apoptosis in cell killing by cisplatin: a flow cytometric study.. Br J Cancer.

[OCR_01255] Rao A. P., Rao P. N. (1976). The cause of G2-arrest in Chinese hamster ovary cells treated with anticancer drugs.. J Natl Cancer Inst.

[OCR_01259] Rusch V., Klimstra D., Venkatraman E., Oliver J., Martini N., Gralla R., Kris M., Dmitrovsky E. (1995). Aberrant p53 expression predicts clinical resistance to cisplatin-based chemotherapy in locally advanced non-small cell lung cancer.. Cancer Res.

[OCR_01266] Sachs L., Lotem J. (1993). Control of programmed cell death in normal and leukemic cells: new implications for therapy.. Blood.

[OCR_01270] Salmon M., Pilling D., Borthwick N. J., Viner N., Janossy G., Bacon P. A., Akbar A. N. (1994). The progressive differentiation of primed T cells is associated with an increasing susceptibility to apoptosis.. Eur J Immunol.

[OCR_01281] Schwartz P. M., Barnett S. K., Atillasoy E. S., Milstone L. M. (1992). Methotrexate induces differentiation of human keratinocytes.. Proc Natl Acad Sci U S A.

[OCR_01288] Smith G. K., Duch D. S., Dev I. K., Kaufmann S. H. (1992). Metabolic effects and kill of human T-cell leukemia by 5-deazaacyclotetrahydrofolate, a specific inhibitor of glycineamide ribonucleotide transformylase.. Cancer Res.

[OCR_01295] Sorenson C. M., Barry M. A., Eastman A. (1990). Analysis of events associated with cell cycle arrest at G2 phase and cell death induced by cisplatin.. J Natl Cancer Inst.

[OCR_01300] Stewart B. W. (1994). Mechanisms of apoptosis: integration of genetic, biochemical, and cellular indicators.. J Natl Cancer Inst.

[OCR_01309] Sun X. M., Cohen G. M. (1994). Mg(2+)-dependent cleavage of DNA into kilobase pair fragments is responsible for the initial degradation of DNA in apoptosis.. J Biol Chem.

[OCR_01318] Tornaletti S., Pfeifer G. P. (1995). Complete and tissue-independent methylation of CpG sites in the p53 gene: implications for mutations in human cancers.. Oncogene.

[OCR_01323] Tounekti O., Pron G., Belehradek J., Mir L. M. (1993). Bleomycin, an apoptosis-mimetic drug that induces two types of cell death depending on the number of molecules internalized.. Cancer Res.

[OCR_01328] Vaux D. L., Strasser A. (1996). The molecular biology of apoptosis.. Proc Natl Acad Sci U S A.

[OCR_01332] Walker P. R., Weaver V. M., Lach B., LeBlanc J., Sikorska M. (1994). Endonuclease activities associated with high molecular weight and internucleosomal DNA fragmentation in apoptosis.. Exp Cell Res.

[OCR_01342] Watanabe H., Kanbe K., Shinozaki T., Hoshino H., Chigira M. (1995). Apoptosis of a fibrosarcoma induced by protein-free culture involves DNA cleavage to large fragments but not internucleosomal fragmentation.. Int J Cancer.

[OCR_01347] Weis M., Schlegel J., Kass G. E., Holmström T. H., Peters I., Eriksson J., Orrenius S., Chow S. C. (1995). Cellular events in Fas/APO-1-mediated apoptosis in JURKAT T lymphocytes.. Exp Cell Res.

[OCR_01352] Wyllie A. H. (1987). Apoptosis: cell death in tissue regulation.. J Pathol.

[OCR_01353] Zhivotovsky B., Wade D., Gahm A., Orrenius S., Nicotera P. (1994). Formation of 50 kbp chromatin fragments in isolated liver nuclei is mediated by protease and endonuclease activation.. FEBS Lett.

[OCR_01090] da Silva C. P., de Oliveira C. R., da Conceiço M., de Lima P. (1996). Apoptosis as a mechanism of cell death induced by different chemotherapeutic drugs in human leukemic T-lymphocytes.. Biochem Pharmacol.

